# Mapping a Large Number of QTL for Durable Resistance to Stripe Rust in Winter Wheat Druchamp Using SSR and SNP Markers

**DOI:** 10.1371/journal.pone.0126794

**Published:** 2015-05-13

**Authors:** Lu Hou, Xianming Chen, Meinan Wang, Deven R. See, Shiaoman Chao, Peter Bulli, Jinxue Jing

**Affiliations:** 1 Key Laboratory of Agricultural Integrated Pest Management, Institute of Plant Protection, Qinghai Academy of Agriculture and Forestry Sciences, Xining, Qinghai, China; 2 Department of Plant Pathology, Washington State University, Pullman, Washington, United States of America; 3 State Key Laboratory of Crop Stress Biology in Arid Areas and College of Plant Protection, Northwest A&F University, Yangling, Shaanxi, China; 4 US Department of Agriculture, Agricultural Research Service, Wheat Genetics, Quality, Physiology and Disease Research Unit, Pullman, Washington, United States of America; 5 US Department of Agriculture, Agricultural Research Service, Cereal Crops Research, Fargo, North Dakota, United States of America; 6 Department of Crop and Soil Sciences, Washington State University, Pullman, Washington, United States of America; Nanjing Agricultural University, CHINA

## Abstract

Winter wheat Druchamp has both high-temperature adult-plant (HTAP) resistance and all-stage resistance to stripe rust caused by *Puccinia striiformis *f. sp. *tritici* (*Pst*). The HTAP resistance in Druchamp is durable as the variety has been resistant in adult-plant stage since it was introduced from France to the United States in late 1940s. To map the quantitative trait loci (QTL) for stripe rust resistance, an F_8_ recombinant inbred line (RIL) population from cross Druchamp × Michigan Amber was phenotyped for stripe rust response in multiple years in fields under natural infection and with selected *Pst* races under controlled greenhouse conditions, and genotyped with simple sequence repeat (SSR) and single nucleotide polymorphism (SNP) markers. Composite interval mapping (CIM) identified eight HTAP resistance QTL and three all-stage resistance QTL. Among the eight HTAP resistance QTL, *QYrdr*.*wgp-1BL*.*2* (explaining 2.36-31.04% variation), *QYrdr*.*wgp-2BL* (2.81–15.65%), *QYrdr*.*wgp-5AL *(2.27–17.22%) and *QYrdr*.*wgp-5BL*.*2* (2.42–15.13%) were significant in all tests; and *QYrdr*.*wgp-1BL*.*1* (1.94–10.19%), *QYrdr*.*wgp-1DS* (2.04–27.24%), *QYrdr*.*wgp-3AL *(1.78–13.85%) and *QYrdr*.*wgp-6BL*.*2* (1.69–33.71%) were significant in some of the tests. The three all-stage resistance QTL, *QYrdr*.*wgp-5BL*.*1* (5.47–36.04%), *QYrdr*.*wgp-5DL* (9.27–11.94%) and *QYrdr*.*wgp-6BL*.*1 *(13.07-20.36%), were detected based on reactions in the seedlings tested with certain *Pst *races. Among the eleven QTL detected in Druchamp, at least three (*QYrdr*.*wgp-5DL* for race-specific all-stage resistance and *QYrdr*.*wgp-3AL* and *QYrdr*.*wgp-6BL*.*2* for race non-specific HTAP resistance) are new. All these QTL, especially those for durable HTAP resistance, and their closely linked molecular markers could be useful for developing wheat cultivars with durable resistance to stripe rust.

## Introduction

Wheat (*Triticum aestivum* L.) is one of the most important crops worldwide, and one of its most damaging diseases is stripe rust, caused by the fungus *Puccinia striiformis* f. sp. *tritici* (*Pst*) [1–3]. Infection by *Pst* on wheat plants can occur anytime from the one-leaf stage to prior to plant maturity [1]. Fungicide application can control stripe rust. However the use of fungicides adds extra cost for wheat production; can have adverse effects on the environment; and may not be fully effective if not applied on time. Growing cultivars with genetic resistance is the most effective, economical, easy to use and environment-friendly way to control the disease [4], [5].

Stripe rust resistance can be classified as race specific resistance and race non-specific resistance based on specificity, and also can be classified as all-stage resistance (also known as seedling resistance) and adult plant resistance (APR) based on the plant growth stage [1], [4], [6]. All-stage resistance, usually race specific and complete, can be detected at seedling stage and protects plants throughout the entire growth cycle when effective against races. However, new virulent races may overcome race specific resistance. In contrast, APR, usually non-race specific, expresses at adult-plant stage. High-temperature adult-plant (HTAP) resistance is further characterized by effectiveness at late stages of plant growth when the weather becomes warm. It is important to know if the APR in a particular cultivar is sensitive to temperature or not, as the information is useful for determining where and under which weather conditions the APR can be effective or not effective, and also for understanding if it is race specific or race non-specific. The level of HTAP resistance is often incomplete and is affected by the plant growth stage, temperature, humidity and inoculum load [1], [4], [7], [8]. Cultivars with HTAP resistance are susceptible at seedling stage if they do not have effective all-stage resistance, but express resistance at adult-plant stage usually after jointing stage. Many wheat cultivars have all-stage and/or HTAP resistance. Identification and mapping of genes conferring HTAP and all-stage resistance can allow breeders to combine different types of resistance into single genetic backgrounds to achieve complete and long-lasting protection of cultivars from stripe rust [1], [4], [5].

Druchamp (PI 174622) is a soft white winter wheat cultivar developed in Ville-de-Paris, France in 1940 and introduced to the US Pacific Northwest for production in 1949 (http://www.ars-grin.gov/cgi-bin/npgs/acc/display.pl?1151600). Although Druchamp is no longer grown for commercial production because other cultivars have higher yields, it has been used to monitor virulence in the *Pst* population. Because of its race specific all-stage resistance, Duchamp has been used in seedling stage to differentiate *Pst* races since 1969 [9]. Later, Druchamp was also found to have high level HTAP resistance [10–13]. A previous monosomic study located three genes for race-specific all-stage resistance to chromosomes 1B, 5B and 6A [14], but the chromosomal locations of these genes have not been determined. Using a biometric approach, three genes were estimated to confer HTAP resistance in Druchamp and significant additive and dominant components and complex gene interactions were observed among resistance gene loci [12], [13]. However, none of the HTAP resistance genes in Druchamp had been mapped to chromosomes prior to the present study.

The objectives of the present study were to map genes or quantitative trait loci (QTL) for either all-stage or HTAP resistance using molecular markers and to assess the specificity or stability of the effects of identified QTL for different types of resistance across multiple environments or tested with different races. This study is expected to generate information vital for understanding durability of HTAP resistance and identify markers that can be used to incorporate HTAP resistance genes from Druchamp into new wheat cultivars.

## Materials and Methods

### Ethics statement

No permits were necessary to conduct reported field experiments, because *Pst* is a naturally occurring plant pathogen in the reported environments and no exotic cultures of the pathogen were used. Research was conducted on land owned by Washington State University. No protected species were sampled. No animal subjects were used in described research. All experiments reported in this manuscript comply with all federal, state and university rules and regulations.

### Plant and pathogen materials

Druchamp (resistant parent) used as the female parent was crossed with susceptible winter wheat Michigan Amber, and 94 F_8_ recombinant inbred lines (RILs) were obtained from 94 F_2_ plants of a single F_1_ plant of the cross through single-seed descent [12], [13]. The RILs were phenotyped in fields under natural *Pst* infection and in greenhouses with selected races, and also genotyped with DNA markers. Seven *Pst* races, PST-25, PST-29, PST-35, PST-45, PST-100, PST-114 and PST-127, were chosen based on the reactions of Druchamp to these races and their virulence formulae and predominance in different periods over the past 40 years [1], [9], [15], [16].

### Greenhouse tests for race specific all-stage resistance

Seedling tests were conducted under controlled conditions in a greenhouse as previously described [10], [11]. About 10 seeds of each line were planted in a 7×7×7 cm pot filled with soil mixture and grown in a rust-free greenhouse. Two-leaf stage seedlings were inoculated with urediniospores of a selected race. Five (PST-29, PST-35, PST-45, PST-100 and PST-114) of the seven selected races were used in the seedling tests as they are avirulent to Druchamp and virulent to Michigan Amber [1], [9], [16]. The inoculated plants were kept in a dew chamber for 24 h at 10^°^C without light, and then grown in a growth chamber using a low diurnal temperature cycle gradually changing between 4^°^C at 2:00 am and 20^°^C at 2:00 pm with 16 h light/8 h dark [10], [11]. A set of wheat varieties that were used to differentiate *Pst* races was included in each race test to confirm the identity of the race [15], [16]. Infection type (IT) based on the 0–9 scale [9] was scored for each line 18 to 21 days after inoculation when stripe rust was fully developed on Michigan Amber.

### Greenhouse tests for HTAP resistance

Adult-plants of the F_8_ RILs and parents were evaluated for studying HTAP resistance at high temperatures in the greenhouse with two (PST-25 and PST-127) of the seven selected races, as the two races are virulent on seedlings of Druchamp and both seedling and adult plants of Michigan Amber, but not virulent to adult plants of Druchamp. In addition, PST-25 and PST-127 represent predominant races in 1980s and recent years, respectively [9], [16]. One-leaf stage seedlings were vernalized in a growth chamber at 2–5^°^C for 40 days. After vernalization, twelve seedlings for each line and each race test were transplanted in three pots of 15-cm in diameter filled with soil mixture. The pots were arranged using a completely randomized block design and grown in a rust-free greenhouse. Plants were inoculated at the heading stage, kept in a dew chamber for 24 h at 10^°^C without light, and then grown in a growth chamber under conditions similar to the seedling tests, except at a higher diurnal temperature cycle gradually changing between 10^°^C at 2:00 am and 30^°^C at 2:00 pm [4], [12]. Data of IT and disease severity (DS, percentage of diseased foliage) were scored as average for each plant 20–22 days after inoculation.

### Field tests

The RIL population and parents were evaluated for stripe rust response in the field nurseries at Pullman, WA (46.7333^°^ N, 117.1667^°^ W, 778 m) in 2006, 2010 and 2011 and Mt. Vernon, WA (48.4200^°^ N, 122.3261^°^ W, 55 m) in 2005, 2010 and 2011. The two locations are about 500 km apart and have different *Pst* race compositions and climatic conditions. In each field experiment, the F_8_ RIL population and parents were planted in a randomized complete block design with three replications. About 30 seeds for each RIL or parent were planted in a 60-cm row with 20 cm between rows. Susceptible variety PS 279 were planted every 20 rows throughout the field to increase the speed and uniformity of stripe rust development. The cultural practices commonly used in wheat production of these regions were performed for fertilization and weed control. All field experiments were conducted under natural infection of *Pst* to allow the evaluation of responses to various races since the natural occurrence of the disease is adequate [1], [17]. Data of IT and DS were visually recorded for each row three times at the heading, flowering and milk stages at Pullman and at the jointing, heading and milk stages at the Mt. Vernon location when Michigan Amber had approximately 50, 80 and >95% DS, respectively. The three-time DS values were used to calculate the value of relative area under the disease progress curve (rAUDPC) for each RIL as previously described [12], [18]. Both the rAUDPC and IT data were used in QTL mapping.

### DNA extraction

Genomic DNA was extracted from the leaf samples using the CTAB method [19]. DNA concentrations were determined using agarose gel electrophoresis [20] and spectrophotometry (NanoDrop ND-1000, Thermo Scientific, Wilmington, DE, USA).

### SSR marker analysis

More than 700 SSR markers were screened to identify those polymorphic between the parents. The polymorphic markers were used to genotype the RILs. The distributions of the markers on the 21 wheat chromosomes were determined based on Somers et al. [21] and the GrainGenes database (http://www.wheat.pw.usda.gov). The M13 tail (5’-CACGACGTTGTAAAACGAC) was added to the 5’ end of each forward primer to detect polymerase chain reaction (PCR) products through direct labeling [22]. The M13 universal primers were labeled with one of the fluorescent dyes FAM (blue), VIC (green), NED (yellow) and PET (red) (Applied Biosystems, Foster City, CA, USA) for detecting different PCR products. Each 12-μl PCR reaction mix contained 4.56 μl ddH_2_O, 1.2 μl Mg-free 10x PCR reaction buffer, 0.48 μl 25 mM MgCl_2_, 0.96 μl 2.5 mM dNTP, 0.06 μl 10 μM M13-tailed forward primer, 0.3 μl 10 μM reverse primer, 0.24 μl 10 μM M13 labeled with appropriate fluorophores dyes (Applied Biosystems, Foster City, CA), 0.2 μl of *Taq* DNA polymerase (5 U/μl) (New England Biolabs, Ipswich, MA) and 4 μμl template DNA (25 ng/μl). PCR was performed in an iCycler (BioRad) thermal cycler (Watertown, MA, USA), using the following conditions: 94^°^C for 5 min hot start, 35–41 cycles (depending upon primers) of 94^°^C for 30 s denaturing, 52 to 61^°^C (depending upon primers) for 45 s annealing and 72^°^C for 1 min, followed by a 10 min final extension at 72^°^C.

PCR products with four different fluorescent dyes were pooled together including 3, 3, 4 and 6 μl of FAM, VIC, NET and PET, respectively and added ddH_2_O to 25 μl, from which 3 μl was transferred into a new tube. A volume of 9 μl Hi- Di Formamide and 1 μl 445-bp Cassul DNA ladder (Applied Biosystems) were added to the tube, giving a total of 13 μl. After denaturing at 95°C for 5 min, the 13 μl mixture was subjected to capillary electrophoresis using an ABI3730 DNA Analyzer (Applied Biosystems, Foster City, CA). Marker alleles were scored using software GeneMapper v1.5 (Softgenetics, State College, PA, USA).

### SNP marker analysis

The RIL population and parents were genotyped with single nucleotide polymorphism (SNP) markers using the Illumina Infinium assay and the Wheat SNP 9K iSelect BeadChips developed by the International Wheat SNP Consortium [23]. SNP genotyping was performed on BeadStation and iScan instruments at the USDA-ARS Biosciences Research Laboratory, Fargo, ND, USA. The raw SNP data were processed with the Illumina GenomeStudio v2011.1 software (Ilumina Inc, San Diego, CA).

### Map construction and QTL analysis

The SSR and SNP data were used to construct the linkage groups using JoinMap version 4.0 [24]. Genetic distances were calculated using the Kosambi mapping function [25]. For each linkage group, the SSR marker order and the assignment to chromosomes were based on the wheat maps published by Somers et al. [21], Sourdille et al. [26] and the GrainGenes database (http://wheat.pw.usda.gov). The chromosomal positions of SNP markers were determined using the genetic maps developed by Cavanagh et al. [23]. The MapChart computer program [27] was used to draw the linkage maps.

For the QTL analysis, different locations and different years of the same location were considered as different environments as the weather conditions, the time and speed of stripe rust development and the race compositions could be different from location to location and from year to years. QTL analysis was performed using mean rAUDPC and IT values of each environment, three-year (2006, 2010 and 2011) means of Pullman, three-year (2005, 2010 and 2011) means of Mt. Vernon experiments and also means of all six environments. QTL mapping was conducted using the composite the interval mapping (CIM) program [28], [29] in the WinQTL Cartographer v2.5 software [30]. The likelihood odds (LOD) thresholds for determining statistically significant QTL were calculated by 1,000 permutations [31]. Based on the permutation tests, LOD 3.0 was set as the threshold to determine HTAP resistance QTL and 5.0 to determine all-stage resistance QTL. A walk speed of 0.5 cM was used for all QTL detections. LOD, additive effects (*a*) and phenotypic coefficients of determination (*R* ^*2*^) for individual QTL were calculated using CIM [32].

### Statistical analysis of phenotypic data

Analysis of variance (ANOVA) was performed using the rAUDPC and IT data to determine the effects of genetic and environmental factors and their interaction using the SAS statistics package (SAS Institute, Inc., Cary, NC, USA). The PROC GLM procedure was used to test lines as a fixed effect, and environments including combination of locations and years and replicates as random effects. The variance components were determined based on ANOVA for a random model generated from PROC GLM. The broad-sense heritability (*H* ^*2*^) was estimated based on the formula *H* ^*2*^ = σ^2^_g_ / σ^2^_p_, where σ^2^_g_ is the genetic variance and σ^2^_p_ represents the phenotypic variance. The genetic variance (σ^2^_g_) was calculated from (σ^2^_L_ - σ^2^_E_)/r, where σ^2^_L_ is the mean variance of the RILs, σ^2^_E_ is the error variance and r equals the number of replications [33]. The correlation coefficients were calculated for pairwise comparison of the population responses in the six environments.

## Results

### Race specific all-stage resistance

When tested with the five races avirulent on Duchamp at the seedling stage and low temperature cycle, Druchamp was resistant (IT 2) and Michigan Amber was susceptible (IT 8) (Table 1). The F_8_ RIL population showed a continuous segregation with various IT. Arbitrary classification of the lines into resistant (IT 2–5) and susceptible (IT 6–8) groups suggested the presence of one gene for resistance in Druchamp to races PST-29 and PST-114, and two genes for resistance to PST-35, PST-45 and PST-100 (Table 1). In each test, not all of the RILs were highly resistant (IT 2) or highly susceptible (IT 8), indicating that the race-specific all-stage resistance was mainly quantitative. Thus, QTL analysis was suitable for mapping the genes conferring the race-specific all-stage resistance in Druchamp.

**Table 1:** Seedling infection types (ITs) of the parents and number of F_8_ recombinant inbred lines (RILs) from the Druchamp × Michigan Amber-derived recombinant inbred lines (RILs) tested in the greenhouse with races of *Puccinia striiformis* f. sp. *tritici*, theoretical segregation ratios of resistant and susceptible lines, and χ^2^ and P values of chi-squared tests for goodness of fit of the observed numbers and expected ratios.

	IT^b^		No. of RILs with IT							Res.^c^	Sus.^c^	Ratio^d^	Chi-squared test^e^	
Race^a^	Druchamp	Michigan Amber	2	3	4	5	6	7	8	(IT 2–5)	(IT 6–8)	(Res.: Sus.)	χ^2^	*P* ^e^
**PST-29**	2	8	31	10	2	6	1	23	21	49	45	1:1	0.17	0.68
**PST-35**	2	8	10	10	1	3	0	19	51	24	70	1:3	0.09	0.77
**PST-45**	2	8	15	12	1	0	24	13	29	28	66	1:3	1.15	0.28
**PST-100**	2	8	6	19	0	0	12	9	48	25	69	1:3	0.06	0.72
**PST-114**	2	8	11	8	3	17	9	5	41	39	55	1:1	2.72	0.10

^a^ Refer to references [1], [9], [15] and [16] for virulence of the races.

^b^ The IT data were recorded based on a 0–9 scale [9] with IT 0–3 as resistant, 4–6 intermediate and 7–9 susceptible.

^c^ Res. = resistant and Sus. = susceptible.

^d^ The 1:1 ratios indicate a single gene and the 1:3 ratio indicate two genes segregated in the RIL population.

^e^ *P* > 0.05 was used for considering the observed numbers of resistant and susceptible RILs fit the theoretical ratio.

### Phenotypic characterization of HTAP resistance

**Fig 1:**
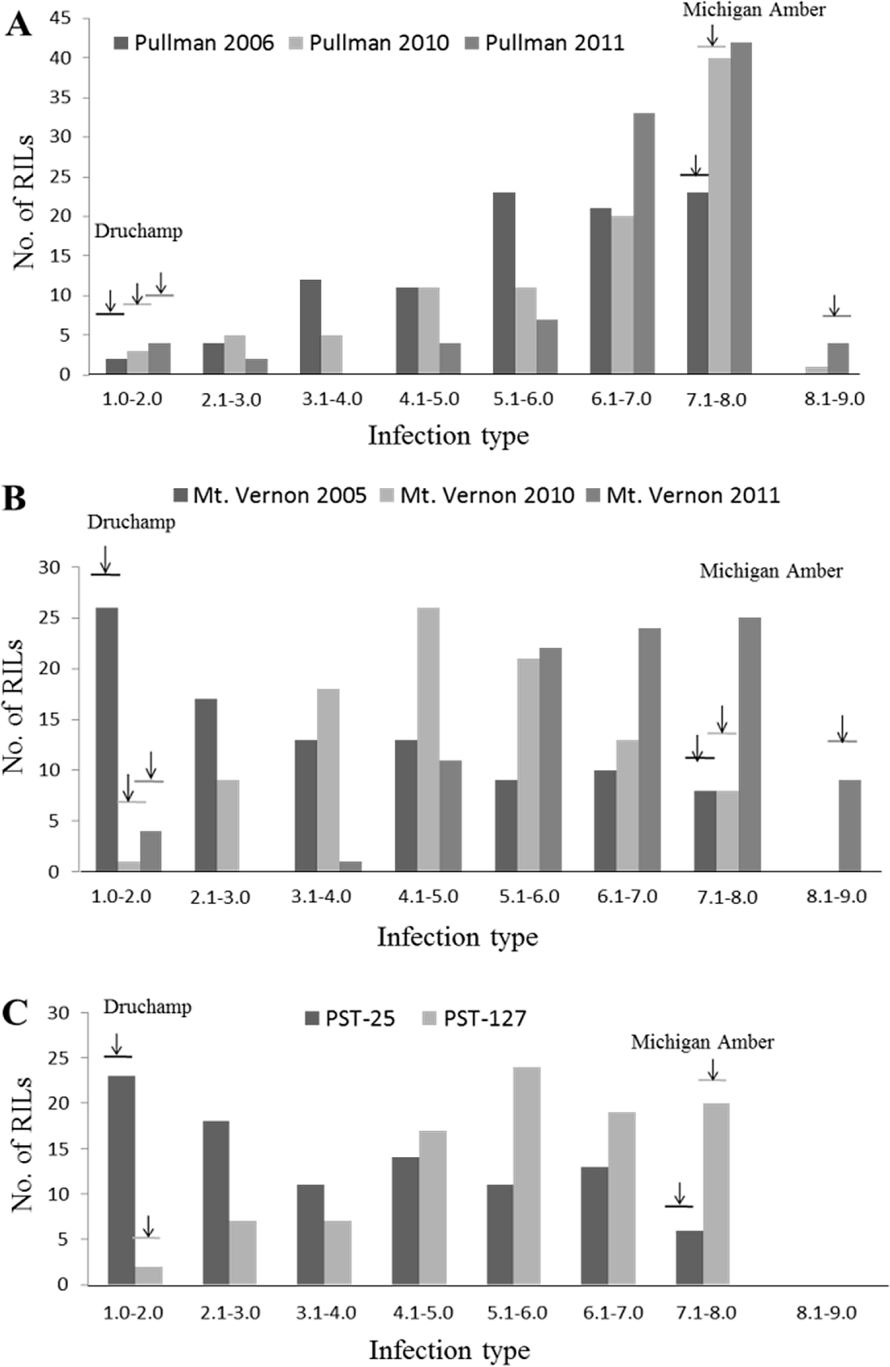
Frequency distributions of mean infection type (IT) values in the Druchamp × Michigan Amber derived recombinant inbred line (RIL) population tested with *Puccinia striiformis* f. sp. *tritici* in various environments. IT distribution of: (**A**) Pullman, WA in 2006, 2010 and 2011; (**B**) Mt. Vernon, WA in 2005, 2010 and 2011; and (**C**) greenhouse with races PST-25 and PST-127.

**Fig 2:**
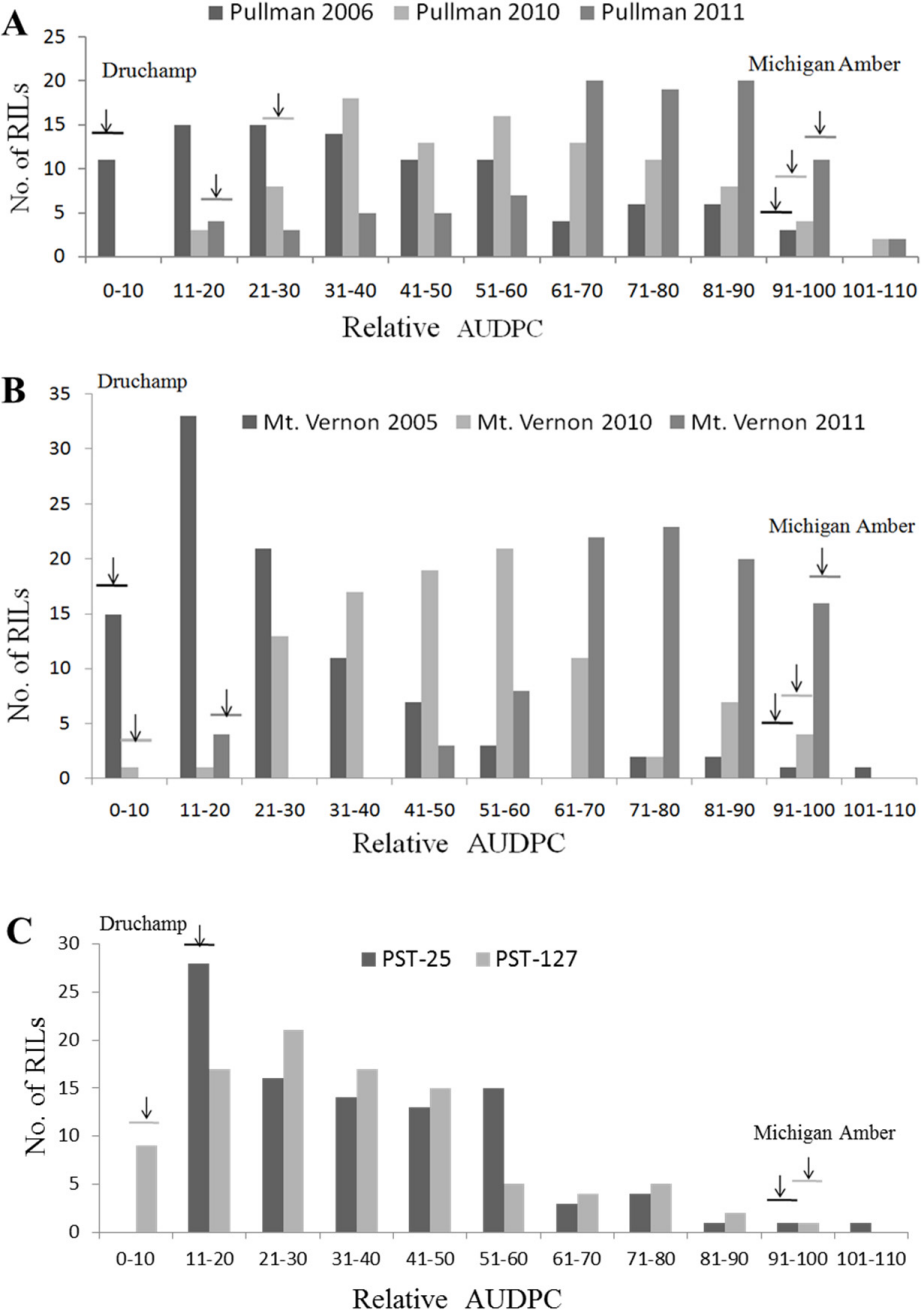
Frequency distributions of mean relative area under the progress curve (rAUDPC) values in Druchamp × Michigan Amber derived recombinant inbred lines tested with *Puccinia striiformis* f. sp. *tritici* in various environments. rAUDPC distribution of: (**A**) Pullman, WA in 2006, 2010 and 2011; (**B**) Mt. Vernon, WA in 2005, 2010 and 2011; and (**C**) greenhouse with races PST-25 and PST-127.

HTAP resistance was evaluated in fields at Pullman in 2006, 2010 and 2011 and at Mt. Vernon in 2005, 2010 and 2011 under natural infection; and under the greenhouse conditions with races PST-25 and PST-127 which are virulent on the seedlings of Druchamp. In all of the experiments, Druchamp was consistently resistant (IT 1–2), whereas Michigan Amber was susceptible (IT 7–9) (Fig 1A, 1B and 1C). In all experiments, stripe rust developed to adequate levels for high quality phenotypic data as Michigan Amber had more than 90% DS at the second or third time of data recording. The mean rAUDPC values of Druchamp ranged from 2.9 to 28.6%, while Michigan Amber had 90–100% mean rAUDPC values in the experiments (Fig 2A, 2B and 2C). Both IT and rAUDPC data of the RIL population showed continuous distributions, indicating that HTAP resistance in Druchamp was quantitatively inherited (Figs 1 and 2).

The ANOVA results showed significant (*P* < 0.0001) genetic variations among RILs for both rAUDPC and IT in the field and greenhouse experiments (Table 2). No significant variation was detected among the replications within each experiment (*P* = 0.16–0.79). The estimated broad-sense heritability values based on all data sets for rAUDPC and IT ranged from 0.66 to 0.94 (Table 2). Correlation coefficients, ranging from 0.43 to 0.95, for either rAUDPC or IT among the six field environments and two race tests in the greenhouse were all significant (*P* < 0.001) (Table 3), suggesting that the expression of HTAP resistance was consistent across the different environments and against different races.

**Table 2:** Analysis of variance and estimates of broad-sense heritabilities (H^2^) of relative area under the disease progress curve (rAUDPC) and infection type (IT) scores of the recombinant inbred lines derived ted from the Druchamp × Michigan Amber cross.

Environment^a^	Source of	rAUDPC				IT			
(Location, year or race)	variation	df	MS	F	*P*	df	MS	F	*P*
**Pullman, 2006**	Line	93	1853.98	14.00	<0.0001	93	7.35	6.50	<0.0001
	Replication	2	107.43	0.97	0.51	2	0.26	0.23	0.79
** **	Error	186	132.44			186	1.13		
	*H* ^*2*^:		0.84				0.90		
**Pullman, 2010**	Line	93	1410.85	5.07	<0.0001	93	7.93	27.93	<0.0001
	Replication	2	197.67	0.59	0.59	2	0.51	1.78	0.17
	Error	186	278.27			186	0.28		
	*H* ^*2*^:		0.67				0.83		
**Pullman, 2011**	Line	93	1362.31	14.46	<0.0001	93	6.34	65.63	<0.0001
	Replication	2	175.43	1.86	0.16	2	0.14	1.48	0.23
	Error	186	94.24			186	0.10		
	*H* ^*2*^:		0.69				0.94		
**Mt. Vernon, 2005**	Line	93	1253.49	18.87	<0.0001	93	11.20	13.65	<0.0001
	Replication	2	84.08	1.27	0.28	2	0.88	1.07	0.35
** **	Error	186	66.43			186	0.82		
	*H* ^*2*^:		0.82				0.80		
**Mt. Vernon, 2010**	Line	93	620.11	36.48	<0.0001	93	3.79	6.51	<0.0001
	Replication	2	17.52	1.03	0.31	2	0.47	0.81	0.37
	Error	186	17.00			186	0.58		
	*H* ^*2*^:		0.92				0.70		
**Mt. Vernon, 2011**	Line	93	925.92	23.34	<0.0001	93	6.61	24.60	<0.0001
	Replication	2	39.57	1.00	0.37	2	0.17	0.63	0.22
** **	Error	186	39.68			186	0.27		
	*H* ^*2*^		0.88				0.93		
**Greenhouse, PST-25**	Line	93	1312.99	31.00	<0.0001	93	11.40	13.13	<0.0001
	Replication	2	27.08	0.64	0.53	2	0.99	1.14	0.32
	Error	186	42.35			186	0.87		
	*H* ^*2*^:		0.94				0.78		
**Greenhouse, PST-127**	Line	93	875.96	26.90	<0.0001	93	7.60	4.95	<0.0001
	Replication	2	35.03	1.08	0.34	2	0.73	0.66	0.60
	Error	186	32.56			186	1.14		
	*H* ^*2*^:		0.90				0.66		

^a^ The field tests in Pullman (eastern Washington) and Mt. Vernon (western Washington) were conducted under the natural infection of *Puccinia striiformis* f. sp. *tritici*. Greenhouse tests were conducted under high-temperature cycle (10–30^°^C); plants were inoculated at the adult growth stage of booting with races PST-25 and PST-127 that were virulent on seedlings of Druchamp; and all growth stages of Michigan Amber were susceptible to the two races.

**Table 3:** Correlation coefficients (*r*) of mean relative area under the disease progress curve (rAUDPC) and infection type (IT) of the Druchamp × Michigan Amber-derived recombinant inbred lines tested in the eight environments.

Environment ^a^	*r* values based on rAUDPC (IT)^b^						
(Location, year or race)	(1)	(2)	(3)	(4)	(5)	(6)	(7)
**(1) Pullman, 2006**							
**(2) Pullman, 2010**	0.62 (0.64)						
**(3) Pullman, 2011**	0.52 (0.55)	0.67 (0.89)					
**(4) Mt. Vernon, 2005**	0.66 (0.67)	0.53 (0.42)	0.49 (0.44)				
**(5) Mt. Vernon, 2010**	0.69 (0.73)	0.57 (0.56)	0.56 (0.52)	0.83 (0.85)			
**(6) Mt. Vernon, 2011**	0.52 (0.58)	0.61 (0.73)	0.82 (0.85)	0.48 (0.47)	0.54 (0.57)		
**(7) GH, PST-25**	0.70 (0.61)	0.61 (0.42)	0.56 (0.42)	0.95 (0.88)	0.85 (0.74)	0.52 (0.43)	
**(8) GH, PST-127**	0.58 (0.51)	0.67 (0.79)	0.89 (0.79)	0.55 (0.50)	0.58 (0.58)	0.71 (0.67)	0.61 (0.52)

^a^ The field tests in Pullman (eastern Washington) and Mt. Vernon (western Washington) were conducted under natural infection of *Puccinia striiformis* f. sp. *tritici*. Greenhouse tests were conducted under high-temperature cycle (10–30^°^C); plants were inoculated at the adult growth stage of booting with races PST-25 and PST-127 that were virulent on seedlings of Druchamp; and all growth stages of Michigan Amber were susceptible to the two races.

^b^ The *r* values based on IT data are given in the parentheses. All of the *r* values were significant at *P* < 0.001.

### Construction of linkage maps

Of the 768 SSR markers screened, 240 (31.3%) were found to be polymorphic between the two parents and were used to test the RIL population. Based on the chi-squared tests, 156 were considered reliable for mapping as they fit the expected 1:1 ratio and thus used in constructing linkage groups. Additionally, a total of 9,000 SNP markers were evaluated on the population, of which 2,535 were found to be polymorphic among the RILs and suitable for linkage construction. Using the 156 SSR and 2,535 SNP markers, 32 linkage groups consisting of 132 SSR and 2,300 SNP markers were constructed, and the remaining 24 SSR markers and 235 SNPs were unlinked based on the LOD 3.0 set by the permutation test. The 32 linkage groups were assigned to 18 wheat chromosomes. Chromosomes 1D, 2B, 3A, 4B, 5A, 5B and 7B each comprised of two linkage groups; 1A and 6A each had three linkage groups; chromosome 7A had four linkage groups, and chromosomes 1B, 2A, 3B, 4A, 5D, 6B, 6D and 7D each had one linkage group. No linkages were found for chromosomes 2D, 3D and 4D. The final linkage groups were assigned to their respective chromosomes using the maps developed Somers et al. [21], Sourdille et al. [26] and the GrainGenes database (http://wheat.pw.usda.gov) for SSR markers and the maps for SNPs developed by Cavanagh et al. [23].

### Mapping of QTL for race-specific all-stage resistance

Because the RILs had various IT values, which did not form distinct resistance and susceptible classes, the IT data were used in QTL mapping. The CIM analysis of each phenotypic dataset of the five race tests revealed the presence of three resistance QTL on the long arm of chromosomes 5B, 5D and 6B. The 5BL QTL (*QYrdr*.*wgp-5BL*.*1*) was detected with races PST-29, PST-45 and PST-100; the 5DL QTL (*QYrdr*.*wgp -5DL*) was detected with PST-35 and PST-45; and the 6BL QTL (*QYrdr*.*wgp -6BL*.*1*) was detected with PST-35, PST-100 and PST-114 (Table 4). All three QTL were contributed by Druchamp. The numbers of the QTL was consistent with those determined through classic genetic analysis by arbitrarily classifying the RILs based on their IT for all five race tests (Table 1). The various infection types observed among the RILs indicated effects of mostly additive and some epistatic interactions. The QTL analysis detected more significant effects of additive (*P* < 0.001) then epistatic interactions (*P* = 0.05) when two QTL were detected.

**Table 4:** Quantitative trait loci for stripe rust resistance detected in the Druchamp × Michigan Amber-derived recombinant inbred line population tested in greenhouse with races *Puccinia striiformis* f. sp. *tritici* at seedling stage and the low-temperature cycle.

QTL	Race	Closest marker	LOD^a^	AE^b^	*R* ^*2*^ (%)^c^
***QYrdr*.*wgp -5BL*.*1***	PST-29	*IWA6271*	12.89	-1.57	36.04
	PST-45	*IWA6271*	6.15	-1.30	30.57
	PST-100	*IWA6271*	2.54	-0.58	5.47
***QYrdr*.*wgp -5DL***	PST-35	*IWA8331*	5.14	-0.80	11.94
	PST-45	*IWA8331*	3.27	-0.71	9.27
***QYrdr*.*wgp -6BL*.*1***	PST-35	*IWA3297*	5.57	-0.86	13.07
	PST-100	*IWA3297*	7.21	-1.09	20.36
	PST-114	*IWA3297*	7.11	-1.00	18.66

^a^ LOD = logarithm (base 10) of odds.

^b^ AE = additive effect. A negative value indicates that the resistance allele for rust reduction is from Druchamp.

^c^ An R^2^ value measured as the percentage of the total observed variation explained indicate the effect of the QTL.

**Fig 3:**
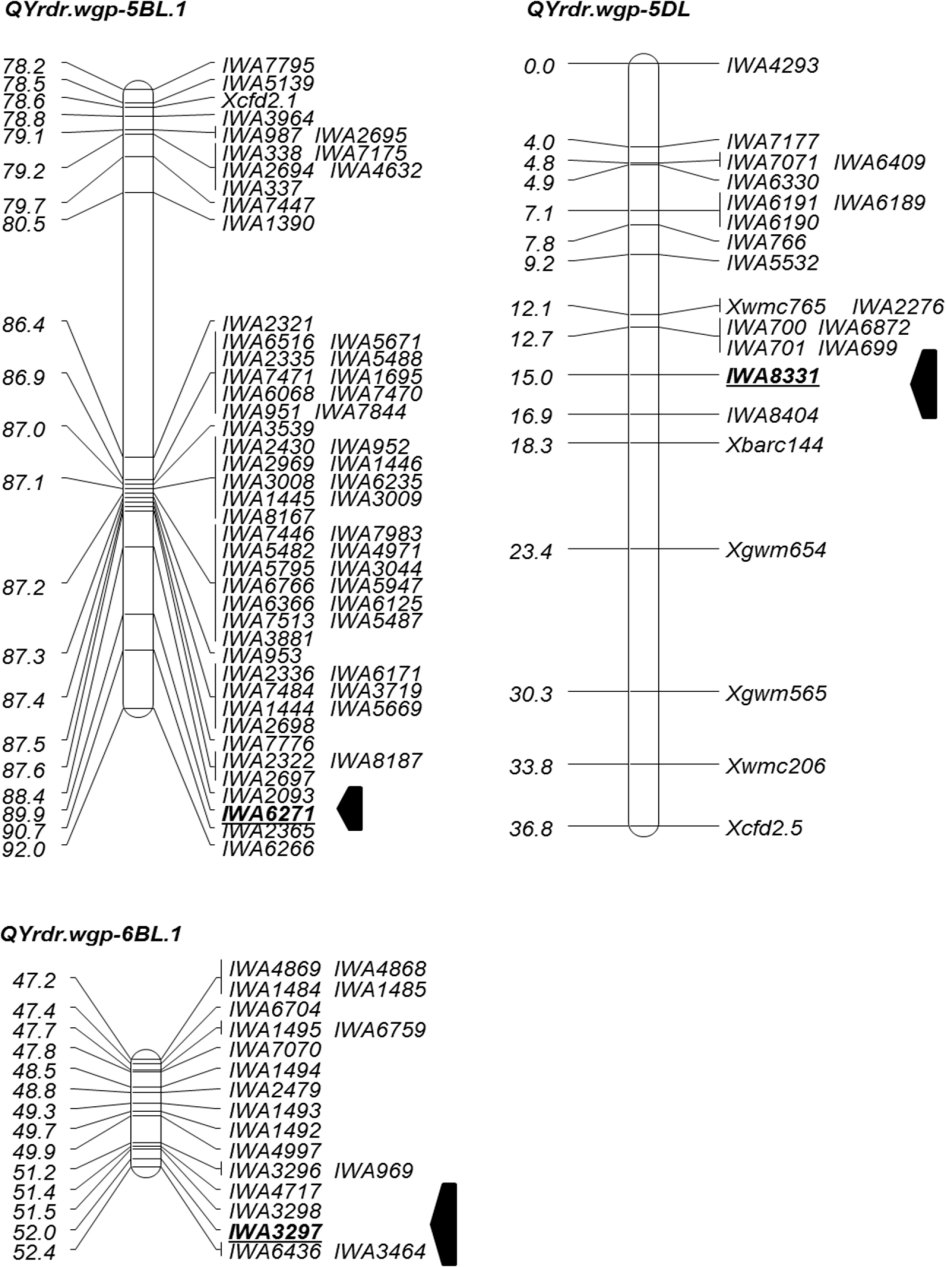
Linkage maps for race-specific all-stage resistance QTL *QYrdr*.*wgp-5BL*, *QYrdr*.*wgp-5D* and *QYrdr*.*wgp-6BL*.*1* constructed using the stripe rust phenotypic data obtained in the greenhouse seedling tests with different *Puccinia striiformis* f. sp. *tritici* races and simple sequence repeat (SSR) and single nucleotide polymorphism (SNP) markers of the recombinant inbred lines of Druchamp × Michigan Amber. Markers with prefix *X* and SSR markers and those with prefix *IWA* are SNPs markers. The locations of the QTL are indicated by the arrows and the markers in bold.

*QYrdr*.*wgp-5BL*.*1*, most closely linked to SNP marker *IWA8581*, explained 36.04, 30.57 and 5.47% of the phenotypic variations in the tests with PST-29, PST-45 and PST-100, respectively (Fig 3; Table 4). *QYrdr*.*wgp-5DL*, close to *IWA8331*, explained 11.94% in the test with PST-35 and 9.27% with PST-45. *QYrdr*.*wgp-6BL*.*1*, linked with *IWA3297*, explained 13.07% (PST-35), 20.36% (PST-100) and 18.66% (PST-114) variations.

### Mapping of QTL for HTAP resistance

**Fig 4:**
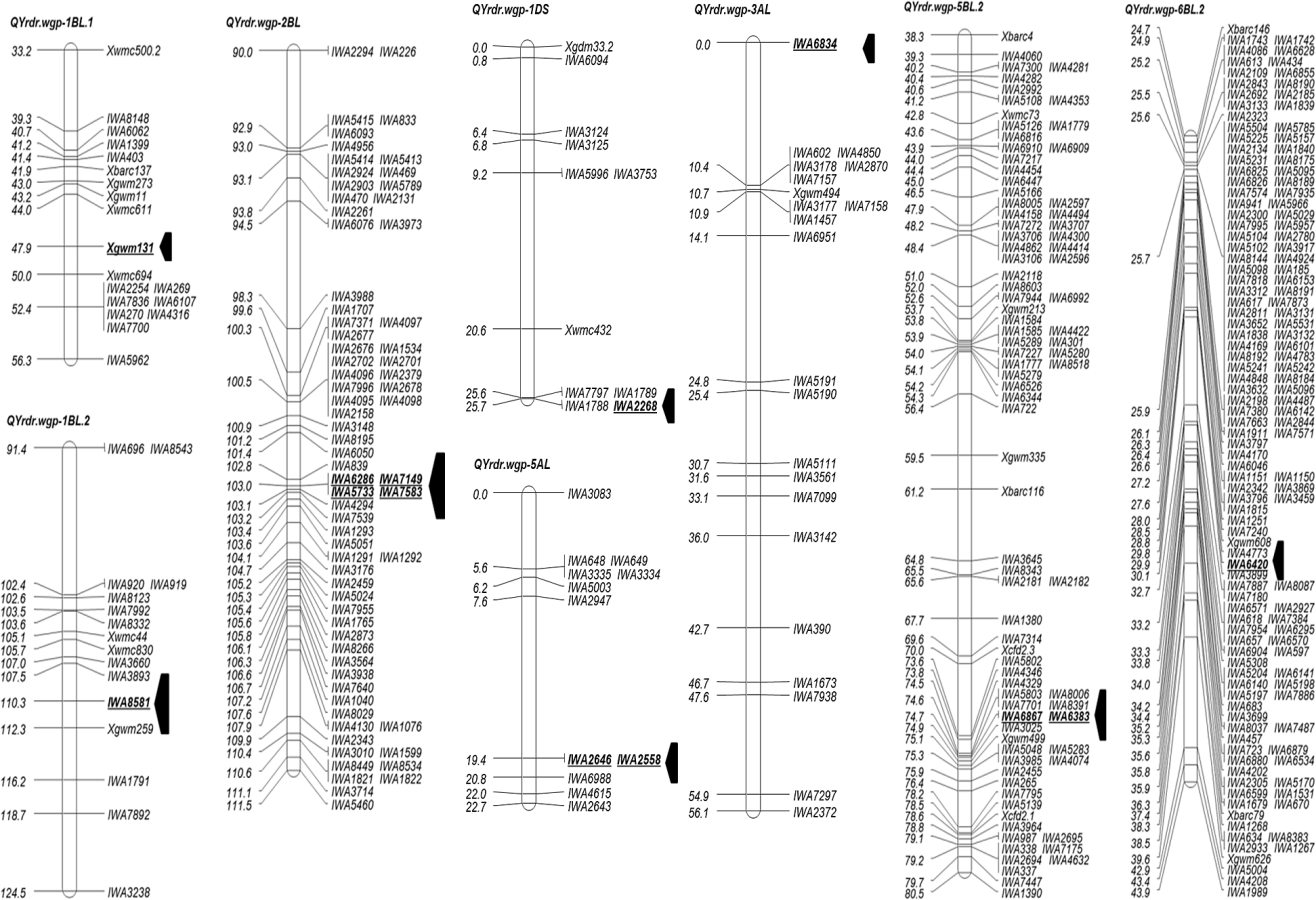
Linkage maps for high-temperature adult-plant (HTAP) resistance eight QTL identified in the Druchamp × Michigan Amber derived recombinant inbred line population using stripe rust phenotypic data obtained under various environments and genotypic data of simple sequence repeat (SSR) and single nucleotide polymorphism (SNP) markers. A total of eight linkage maps were identified. Fig 4.1 shows QTL *QYrdr*.*wgp-1BL*.*1*, *QYrdr*.*wgp-1BL*.*2* and *QYrdr*.*wgp-2BL*; Fig 4.2 shows *QYrdr*.*wgp-1DS*, *QYrdr*.*wgp-3AL*, and *QYrdr*.*wgp-5AL*; and Fig 4.3 shows *QYrdr*.*wgp-5BL*.*2* and *QYrdr*.*wgp-6BL*.*2*. Markers with prefix *X* are SSR markers and those with prefix *IWA*) are SNPs. The locations of the QTL are indicated by the arrows and the markers in bold.

Eight QTL for HTAP resistance were detected using the IT and rAUDPC data of the six field experiments, combined mean IT and rAUDPC data of the six field experiments and the two race greenhouse tests. Two of the QTL were mapped on chromosomes 1BL and one each on 1DS, 2BL, 3AL, 5AL, 5BL and 6BL (Fig 4; Tables 5 and 6). These QTL were designated as *QYrdr*.*wgp-1BL*.*1*, *QYrdr*.*wgp-1BL*.*2*, *QYrdr*.*wgp-1DS*, *QYrdr*.*wgp-2BL*, *QYrdr*.*wgp-3AL*, *QYrdr*.*wgp-5AL*, *QYrdr*.*wgp-5BL*.*2* and *QYrdr*.*wgp-6BL*.*2*, respectively. All QTL for either rAUDPC or IT were significant and were contributed by Druchamp.

**Table 5:** Quantitative trait loci for stripe rust resistance for the relative area under the disease progress curve (rAUDPC) and infection type (IT) in the Druchamp × Michigan Amber derived recombinant inbred line population in 2006, 2010 and 2011 at Pullman and in 2005, 2010 and 2011 at Mt. Vernon.

	Closest			2005 or 2006 ^b^			2010			2011			Location mean		
QTL^a^	marker	Data	Location	LOD^c^	AE^d^	*R* ^2^ (%)	LOD	AE	*R* ^2^ (%)	LOD	AE	*R* ^2^ (%)	LOD	AE	*R* ^2^ (%)
*QYrdr*.*wgp-1BL*.*1*	*Xgwm131*	rAUDPC	Pullman	4.06	-8.24	10.19	ND^e^	ND	ND	ND	ND	ND	ND	ND	ND
			Mt. Vernon	2.81	-4.88	5.04	4.18	-5.32	6.94	ND	ND	ND	3.27	-3.74	4.66
			Mean^f^										2.90	-4.05	5.23
		IT	Pullman	1.15	-0.26	2.62	ND	ND	ND	1.54	0.27	3.44	ND	ND	ND
			Mt. Vernon	2.16	-0.36	3.33	2.57	-0.26	3.33	ND	ND	ND	1.23	-0.20	1.94
			Mean										ND	ND	ND
***QYrdr*.*wgp-1BL*.*2***	*IWA8581*	rAUDPC	Pullman	11.29	-14.16	31.04	7.62	-9.86	17.22	3.00	-5.19	5.45	9.34	-9.68	21.13
			Mt. Vernon	9.85	-9.72	21.47	12.17	-9.41	22.91	1.11	-2.81	2.36	10.88	-7.99	22.41
			Mean										12.21	-9.32	28.58
		IT	Pullman	ND	ND	ND	1.49	-0.39	5.32	1.59	-0.29	3.67	ND	ND	ND
			Mt. Vernon	6.56	-0.66	11.00	8.38	-0.55	14.47	ND	ND	ND	7.02	-0.54	13.73
			Mean										1.83	-0.26	3.57
*QYrdr*.*wgp-1DS*	*IWA2268*	rAUDPC	Pullman	1.05	-3.61	2.04	ND	ND	ND	ND	ND	ND	ND	ND	ND
			Mt. Vernon	7.31	-7.94	14.71	9.88	-8.24	19.07	1.58	-3.83	4.57	7.21	-6.15	15.69
			Mean										2.18	-3.25	3.48
		IT	Pullman	ND	ND	ND	ND	ND	ND	1.14	-0.24	2.57	ND	ND	ND
			Mt. Vernon	12.80	-0.98	27.24	11.86	-0.66	24.98	1.28	-0.28	3.45	10.23	-0.65	21.93
			Mean										1.98	-0.27	5.84
***QYrdr*.*wgp-2BL***	*IWA7583*	rAUDPC	Pullman	3.92	-7.92	9.15	ND	ND	ND	3.68	-6.02	7.64	3.07	-4.83	6.03
			Mt. Vernon	6.90	-7.79	13.72	9.26	-7.85	15.65	3.68	-5.63	10.19	5.88	-5.43	10.15
			Mean										5.27	-5.66	10.31
		IT	Pullman	1.24	-0.28	2.81	1.21	-0.35	4.32	1.93	-0.32	4.30	2.86	-0.36	6.40
			Mt. Vernon	8.43	-0.75	14.47	7.40	-0.50	12.06	ND	ND	ND	5.87	-0.47	10.80
			Mean										4.04	-0.42	10.14
*QYrdr*.*wgp-3AL*	*IWA6834*	rAUDPC	Pullman	ND	ND	ND	ND	ND	ND	6.23	-8.20	13.85	ND	ND	ND
			Mt. Vernon	ND	ND	ND	1.26	-3.46	2.94	1.90	-3.89	3.97	1.47	-2.69	2.19
			Mean										1.09	-2.49	1.78
		IT	Pullman	5.10	-0.57	12.76	ND	ND	ND	ND	ND	ND	3.25	-0.38	7.28
			Mt. Vernon	ND	ND	ND	ND	ND	ND	2.13	-0.39	5.64	ND	ND	ND
			Mean										4.15	-0.41	9.37
***QYrdr*.*wgp-5AL***	*IWA2558*	rAUDPC	Pullman	ND	ND	ND	3.43	-6.46	7.79	3.42	-5.68	5.97	2.22	-4.17	3.78
			Mt. Vernon	3.18	-5.37	5.80	4.92	-5.76	7.61	2.82	-4.80	7.13	5.19	-5.55	9.38
			Mean										2.02	-3.52	3.24
		IT	Pullman	4.83	-0.59	12.18	4.31	-0.68	15.42	5.56	-0.60	14.90	5.24	-0.61	17.22
			Mt. Vernon	4.01	-0.52	6.22	8.00	-0.55	13.40	ND	ND	ND	5.70	-0.50	10.86
			Mean										6.88	-0.57	16.56
***QYrdr*.*wgp-5BL*.*2***	*IWA6383*	rAUDPC	Pullman	5.19	-9.90	15.13	1.81	-4.18	3.54	ND	ND	ND	3.75	-5.41	7.37
			Mt. Vernon	1.70	-3.43	2.74	2.29	-3.48	3.94	2.85	-3.72	6.28	3.78	-4.92	7.56
			Mean										4.99	-5.42	9.48
		IT	Pullman	1.14	-0.25	2.42	ND	ND	ND	3.87	-0.49	11.26	1.57	-0.25	3.13
			Mt. Vernon	3.61	-0.47	5.69	1.78	-0.24	2.68	3.46	-0.35	6.59	5.41	-0.54	13.29
			Mean										1.87	-0.26	4.21
*QYrdr*.*wgp-6BL*.*2*	*IWA6420*	rAUDPC	Pullman	2.44	-5.87	5.15	4.99	-7.16	10.53	5.08	-7.87	21.63	4.27	-5.71	8.49
			Mt. Vernon	1.42	-3.33	2.49	1.18	-2.78	1.91	4.08	-5.61	10.86	2.63	-3.56	4.30
			Mean										1.53	-3.54	4.08
		IT	Pullman	1.07	-0.28	3.03	4.07	-0.66	15.69	2.32	-0.41	7.58	2.66	-0.39	5.87
			Mt. Vernon	1.17	-0.26	1.69	1.23	-0.21	2.14	2.26	-0.43	8.56	1.47	-0.23	2.54
			Mean										2.19	-0.29	4.48

^a^ QTL in bold were stable and consistently detected in multiple environments.

^b^ The fields tests during the 2005 and 2006 growing seasons were conducted at Mt. Vernon (western Washington) and Pullman (eastern Washington).

^c^ LOD = logarithm (base 10) of odds.

^d^ AE = additive effect. A negative value indicates that the resistance allele for rust reduction is from Druchamp.

^e^ ND = No data.

^f^ Overall mean of the six environments (two locations and three years at each location).

**Table 6:** Quantitative trait loci (QTL) for stripe rust resistance based on relative area under the disease progress curve (rAUDPC) and infection type (IT) data detected in the Druchamp × Michigan Amber-derived recombinant inbred line population at adult plant stage inoculated with races PST-25 and PST-127 of *Puccinia striiformis* f. sp. *tritici* in greenhouse under the high-temperature cycle.

			PST-25			PST-127		
QTL^a^	Marker	Data	LOD^b^	AE^c^	*R* ^2^ (%)	LOD	AE	*R* ^2^ (%)
*QYrdr*.*wgp-1BL*.*1*	*Xgwm131*	rAUDPC	3.11	-5.04	5.34	ND^d^	ND	ND
		IT	2.32	-0.43	4.42	ND	ND	ND
***QYrdr*.*wgp-1BL*.*2***	*IWA8581*	rAUDPC	13.50	-11.72	30.1	2.43	-5.60	6.27
		IT	11.64	-1.06	28.28	2.33	-0.46	7.75
*QYrdr*.*wgp-1DS*	*IWA2268*	rAUDPC	5.62	-6.77	10.29	ND	ND	ND
		IT	6.62	-0.77	15.3	1.57	-0.37	5.33
***QYrdr*.*wgp-2BL***	*IWA7583*	rAUDPC	7.97	-8.56	15.64	1.93	-4.73	5.28
		IT	5.43	-0.68	11.46	3.47	-0.55	11.43
*QYrdr*.*wgp-3AL*	*IWA6834*	rAUDPC	ND	ND	ND	3.72	-7.20	11.82
		IT	1.09	-0.35	3.01	2.37	-0.46	7.92
***QYrdr*.*wgp-5AL***	*IWA2558*	rAUDPC	1.24	-3.42	2.27	1.33	-4.11	3.46
		IT	ND	ND	ND	3.73	-0.65	15.23
***QYrdr*.*wgp-5BL*.*2***	*IWA6383*	rAUDPC	ND	ND	ND	1.51	-4.50	4.53
		IT	ND	ND	ND	2.24	-0.47	8.32
*QYrdr*.*wgp-6BL*.*2*	*IWA6420*	rAUDPC	1.58	-3.28	2.42	4.97	-7.83	14.23
		IT	ND	ND	ND	5.24	-0.94	33.71

^a^ QTL in bold were stable and consistently detected in multiple environments.

^b^ LOD = logarithm (base 10) of odds.

^c^ AE = additive effect. A negative value indicates that the resistance allele for rust reduction is from Druchamp.

^d^ ND = No data.

Among the eight QTL detected for HTAP resistance, *QYrdr*.*wgp-1BL*.*2* was the most consistent and provided the highest level of resistance. It was detected in all six field and two greenhouse experiments, and also detected with Pullman and Mt. Vernon mean data and the overall mean data of all six field experiments (Tables 5 and 6). This QTL explained 2.36–31.04% of the phenotypic variance, depending upon the experiment. The closest marker to the QTL was SNP marker *IWA8581*, and SSR markers *Xwmc830* and *Xgwm259* flanked the locus by approximately 4.6 and 2.0 cM, respectively (Fig 4).

*QYrdr*.*wgp-2BL*, *QYrdr*.*wgp-5AL* and *QYrdr*.*wgp-5BL*.*2* were also stable. They were detected in all six field and two greenhouse experiments, except that *QYrdr*.*wgp-5BL*.*2* was not detected in the greenhouse test with race PST-25. These QTL explained 2.81–15.65, 2.27–17.22 and 2.42–15.13% of the phenotypic variation, respectively (Table 5). The nearest marker to *QYrdr*.*wgp-2BL* was *IWA7583*, *QYrdr*.*wgp-5AL* was *IWA2558* and *QYrdr*.*wgp-5BL*.*2* was *IWA6383* (Fig 4).

*QYrdr*.*wgp-6BL*.*2*, most closely associated with SNP marker *IWA6420* (Fig 4) and explained 1.69–33.71% of the phenotypic variation, was detected in all experiments, but only the LOD values from the experiments of Pullman 2010, Pullman 2011, Mt. Vernon 2011 and race PST-127 in the greenhouse and the Pullman three-year mean data were greater than the threshold value of 3.0, indicating that relatively low stability compared to the other QTL.

*QYrdr*.*wgp-1BL*.*1* was detected in four of the six field experiments, the PST-25 test in the greenhouse and with the Mt. Vernon three-year mean data as well as the mean data of all six-field experiments (Tables 5 and 6). The QTL explained 1.94–10.19% of the observed phenotypic variation. *QYrdr*.*wgp-1BL*.*1* was significantly associated with SSR marker *Xgwm131* and flanked by *Xwmc611* and *Xwmc694* at approximate distances of 3.9 and 2.1 cM, respectively (Fig 4). The nearest SNP marker to *QYrdr*.*wgp-1DS* was *IWA2268* (Fig 4). This QTL was detected in five of the six field experiments and the two tests with races PST-25 and PST-127 in the greenhouse, and with the means from the Mt. Vernon three-year data and the six field-experiment data. It explained 2.04–27.24% of the phenotypic variance (Tables 5 and 6). *QYrdr*.*wgp-3AL* was detected in four of the six field experiments and in the tests with races PST-25 and PST-127 in the greenhouse, and with means of the data from Pullman, Mt. Vernon and both Pullman and Mt. Vernon. This QTL, explaining 1.78–13.85% of the observed phenotypic variation, was closely associated with *IWA6834* (Fig 4; Tables 5 and 6).

### Effect of the number of QTL in combination

**Fig 5:**
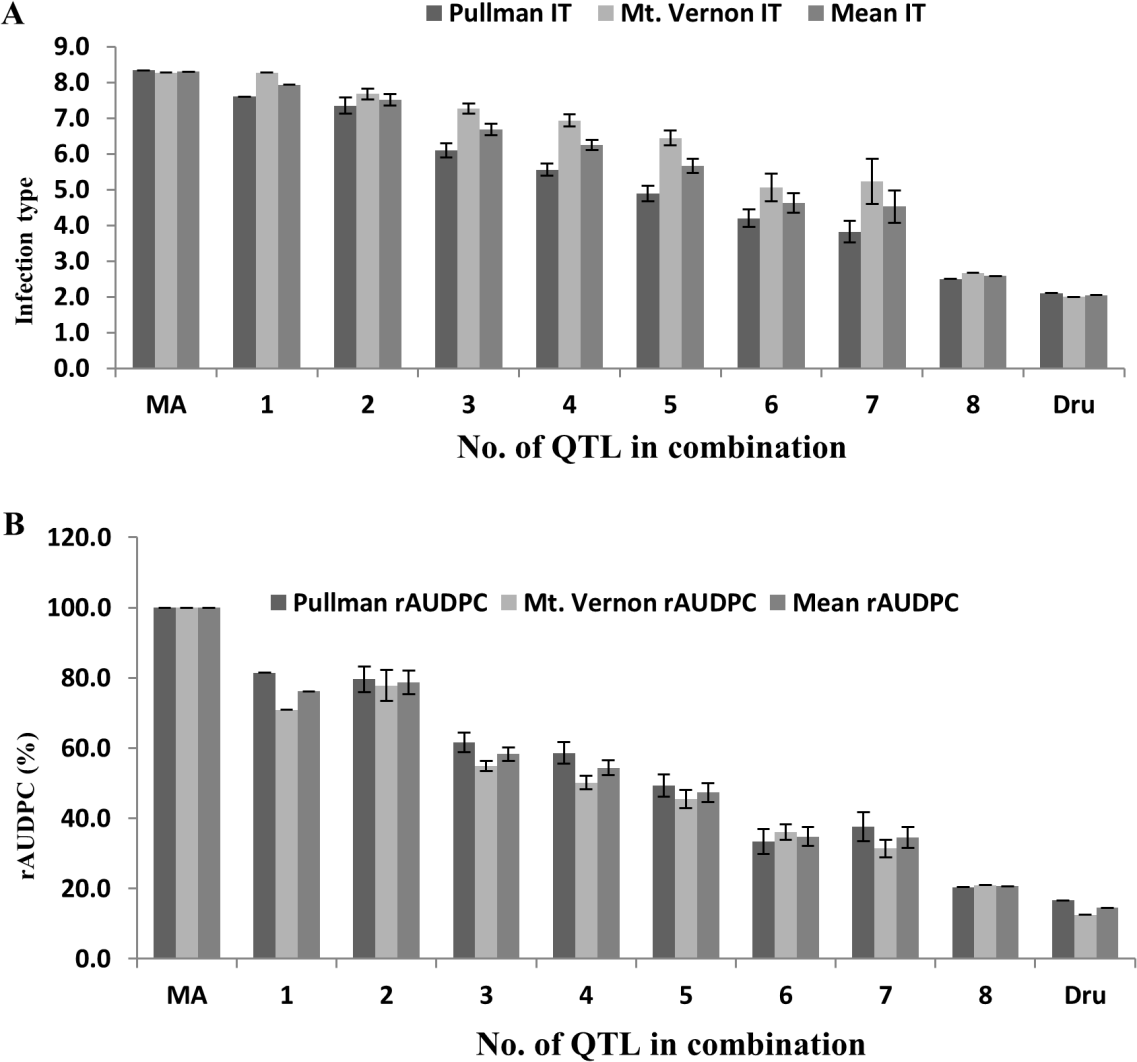
Effects of the number of QTL on high-temperature and adult-plant (HTAP) resistance to stripe rust illustrated by the mean infection type (IT) and relative area under the progress curve (rAUDPC) values of the recombinant inbred lines (RILs) derived from the Druchamp (Dru) × Michigan Amber (MA) in Pullman and Mt. Vernon, showing RILs with more QTL were more resistant. The data of IT are shown in (A) and those of rAUDPC in (B). MA (the susceptible parent does not have any stripe rust resistance QTL and Dru (the resistant parent) has eight QTL for HTAP resistance.

To determine the effects of QTL in various combinations on HTAP resistance, the 94 RILs were classified into genotypic groups based on the presence of markers closely associated with the eight QTL (S1 Table). These genotypes were further grouped into eight groups based on the number of potential QTL for HTAP resistance. Fig 5 shows the differences in the mean IT and rAUDPC values of the eight groups. In general, RILs with more resistance QTL had lower IT and rAUDPC values. This observation is an indication of additive effects of the QTL, with resistance increasing as the number of QTL increases. This is supported by the significant additive effects (*P* < 0.01) obtained by the QTL analysis.

### Interactions between the QTL and environments

The interactions to different environments were determined for each of the HTAP QTL. Significant interactions were detected for *QYrdr*.*wgp-1BL*.*2* (*P* = 0.01 for IT and 0.06 for rAUDPC), *QYrdr*.*wgp-1DS* (*P* = 0.0001 for IT and 0.05 for rAUDPC) and *QYrdr*.*wgp-6BL*.*1* (*P* = 0.06 for IT and 0.02 for rAUDPC). For the other five QTL, the environmental interactions were not significant for either the IT or rAUDPC data (*P* = 0.16 to 0.99). The lack of significant interaction with different environments for the majority of the QTL may explain the durability and consistency of the HTAP resistance against the diverse races that are virulent to the seedlings of Druchamp over the years and in the different locations.

## Discussion

Druchamp has been resistant to stripe rust since its introduction from France to the US more than 60 years ago. Even after the first Druchamp seedling-virulent race, PST-6, was detected in 1974 and subsequently many virulent races were predominant [9], [16], the cultivar has never become susceptible in the field nurseries. Druchamp was determined to have both race-specific all-stage and race non-specific HTAP resistance [10–14]. In the present study, we mapped three QTL for the all-stage resistance and eight QTL for the HTAP resistance in Druchamp. Compared to the previous QTL mapping studies [4], [34], this study reports the highest number of QTL for resistance to stripe rust in a single wheat cultivar.

### *QYrdr*.*wgp-1BL*.*1* and *QYrdr*.*wgp-1BL*.*2*

Two QTL, *QYrdr*.*wgp-1BL*.*1* and *QYrdr*.*wgp-1BL*.*2*, were mapped on chromosome 1BL. Several stripe rust resistance genes have been reported on 1BL, including *QYr*.*jirc-1B* [35], *QYr*.*cimmyt-1BL* [36], *Yr29* [37], *QPst*.*jic-1B* [38], *YrChk* [39], *YrExp1* [40], *QYrex*.*wgp-1BL* [41], *YrN*.*S-2* [42] and *YrV3* [43]. Because *YrChk*, *YrExp1*, *YrN*.*S-2* and *YrV3* confer all-stage resistance, they are likely different from *QYrdr*.*wgp-1BL*.*1* and *QYrdr*.*wgp-1BL*.*2*, which confer HTAP resistance.

*QYrex*.*wgp-1BL*, *QYr*.*jirc-1B*, *QYr*.*cimmyt-1BL*, *QPst*.*jic-1B* and *Yr29* have been reported to confer HTAP resistance. *QYrex*.*wgp-1BL*, a small effect QTL in spring wheat Express, is flanked by SSR marker *Xwmc631* and RGAP marker *Xwgp78* [41]. *QYr*.*jirc-1B*, a minor QTL derived from ‘Fukuho-komugi’, with the closed marker *Xwmc320*, was located in the region near the centromere [35]. *QYr*.*cimmyt-1BL* was mapped between SSR markers *Xgwm140* and *Xgwm259* [36]. Based on the closely linked markers and chromosomal positions, the two QTL for HTAP resistance identified in the present study are likely different from these genes. *QPst*.*jic-1B* was considered the same as *Yr29*, as it mapped to the distal end of 1BL between markers *Xgwm818* and *Xgwm259*, the same position as *Yr29* on 1BL [38]. *Yr29* is flanked by the SSR loci *Xwmc44* and *Xgwm259* [37]. These two markers are also flanking *QYrdr*.*wgp-1BL*.*2* (Fig 4), suggesting that *QYrdr*.*wgp-1BL*.*2* is likely the same gene as *Yr29* or a different allele at that locus. The chromosomal region containing *QYrdr*.*wgp-1BL*.*1* and *QYrdr*.*wgp-1BL*.*2* on 1BL appears to be a hot spot for resistance genes against stripe rust and other diseases.

Druchamp was reported to have all-stage resistance gene *Yr3a*, which was reported on chromosome 1B using monosomic analysis [14]. That gene provides resistance to *Pst* races CDL-1, CDL-21 and CDL-29, which were later renamed as PST-1, PST-21 and PST-29, respectively [1]. In the present study, the gene for resistance to PST-29 was mapped to 5BL (*QYrdr*.*wgp-5BL*.*1*). The two QTL on 1BL, *QYrdr*.*wgp-1BL*.*1* and *QYrdr*.*wgp-1BL*.*2*, were only detected in adult-plant stage, and no all-stage resistance QTL was detected on 1B with the five tested races including PST-29. The present study corrected the chromosomal location of the gene in Druchamp for resistance to PST-29 to be in 5BL, instead of 1B.

### QYrdr.wgp-1DS

*Yr25* was reported on chromosome 1D (Calonnec and Johnson 1998), but its specific chromosomal location is unknown. *Yr25* is a major gene for resistance to some races in other countries [44], but largely ineffective to the *Pst* population in the US [45]. Ren et al. [46] mapped adult plant resistance QTL *QYr*.*caas-1DS* in wheat cultivar Naxos on chromosome 1DS flanked by SSR markers *Xgwm353* and *Xgdm33b*, and *Xwmc432* is close to the QTL region. In the present study, *QYrdr*.*wgp-1DS*.*1* is about 5 cM away from *Xwmc432* in (Fig 4). Thus, these two QTL could be the same or different alleles in the same chromosomal region.

### QYrdr.wgp-2BL

Several adult plant resistance QTL for stripe rust resistance were reported on wheat chromosome 2BL [4], [34], [47], including *QTL 2BL* [48], *QYr*.*csiro-2BL* [49], *QYR1* [50], *QYr*.*inra-2BL* [51], *QYraq*.*cau-2BL* [52] and *QYr*.*caas-2BL* [46]. *QTL 2BL* in wheat cultivar Deben was located between *Xwmc149* and *Xwmc317a* [48]. *QYr*.*csiro-2BL* from Avocet S was found to be flanked by *Xgwm1027* and *Xgwm619* at the distal end of chromosome 2BL [49]. *QYR1* from Camp Remy was located in the marker interval *Xgwm47-Xgwm501* [50]. *QYr*.*inra-2BL*, also from Camp Remy, was located in the marker interval *Xbarc101-Xgwm120* [51]. *QYraq*.*cau-2BL* in cultivar Aquileja was found to be flanked by *Xwmc175* and *Xwmc332* [52]. *QYr*.*caas-2BL* from Naxos was mapped between SSR markers *Xwmc441* and *Xwmc361* in a larger interval [46]. Because *QYrdr*.*wgp-2BL* was mapped with SNP markers (Fig 4), its relationships with the other adult plant resistance QTL on chromosome 2BL could not be determined.

### QYrdr.wgp-3AL

On chromosome 3A, only three stripe rust resistance genes have been reported. *YrTr2* is a gene for race-specific all-stage resistance, and its chromosomal arm is unknown [53]. Lillemo et al. [33] identified a QTL (*QRYr3A*.*1*) on chromosome 3AS linked to SSR marker *Xbarc310* in wheat cultivar Saar. *YrQ2* conferring slow-rusting in wheat line Xichang 76–9 was mapped on chromosome 3AS and linked to SSR markers *Xwmc11* and *Xbarc57* within 12.1 cM [54]. Because *QYrdr*.*wgp-3AL* confers HTAP resistance and is on chromosome 3AL, it should be a new gene for stripe rust resistance.

### QYrdr.wgp-5AL

Several stripe rust resistance genes have been reported on chromosome 5AL [4], [34], [47]. *QYR5*, a minor QTL in Opata 85, was mapped on 5AL with RFLP markers interval *Fbb209–abg391* and SSR marker *Xgwm126* [50]. *Yr34*, which confers an intermediate seedling IT and very low adult plant response in wheat line WAWHT2046, was mapped to the chromosome arm 5AL proximal to the awn inhibitor gene *B1* locus with a marker/gene order of *Xgwm595-Xgwm6a-Xgwm291-Xgwm410*.*2-B1-Yr34* [55]. *QYrtm*.*pau-5A*, from *T*. *boeoticum* accession Pau5088, was mapped on 5AL in an 8.9 cM interval between *Xbarc151* and *Xcfd12* [56]. *QYr*.*caas-5AL*, derived from Chinese landrace Pingyuan 50, was found to be flanked by SSR markers *Xwmc410* and *Xbarc261* [57]. *Yr48*, a gene underlying the partial stripe rust resistance QTL in synthetic derivative wheat PI 610750, was mapped on the distal region of 5AL close to marker *Xcfa2149* [58]. Jagger et al. [59] mapped *QPst*.*jic-5A* in Alcedo on chromosome 5A with SSR markers *Xwmc752* and *Xgwm786*. This QTL is likely in the short arm of chromosome 5A based on the position of *Xwmc752* [21]. Ren et al. [46] mapped adult-plant resistance QTL *QYr*.*caas-5AL*.*2*, derived from wheat line Shanghai 3/Catbird, in the marker interval between *XwPt-1903-5AL* and *Xwmc727-5AL*, in the same chromosomal region as *QYr*.*caas-5AL* reported by Lan et al. [57]. In the present study, *QYrdr*.*wgp-5AL* was mapped with SNP markers, and therefore, its relationships with the genes previously reported on 5AL could not be clearly determined.

### *QYrdr*.*wgp-5BL*.*1* and *QYrdr*.*wgp-5BL*.*2*

In the present study, we mapped two QTL on chromosome 5BL, *QYrdr*.*wgp-5BL*.*1* for race specific all-stage resistance and *QYrdr*.*wgp-5BL*.*2* for HTAP resistances. Worland [60] reported the *Yr3* locus in Nord Desprez and Vilmorin 23 on 5BL. As Druchamp was reported to have *Yr3a* that is effective against race PST-29 [11], [14], *QYrdr*.*wgp-5BL*.*1* should be *Yr3a*. In addition to several SNP markers tightly linked to this QTL, SSR marker *Xcfd2*.*1* is about 11.3 cM proximal to the locus (Fig 3). *Yr88375*, a races-specific all-stage resistance gene in the Chinese wheat line Zhongliang 88375, is closely linked to *Xgdm116* and *Xwmc810* [61]. Based on the positions of these markers on the consensus map [21], *Yr88375* is about 20 cM from *QYrdr*.*wgp-5BL*.*1*.

Several QTL have been previously reported on chromosome 5BL [4], [34], [47]. *QYr*.*jirc-5BL* in the wheat cultivar Oligoculm is linked to SSR marker *Xwmc415* [34]. *QYr*.*inra-5BL*.*1* and *QYr*.*inra-5BL*.*2*, both from Camp Remy, were mapped in marker intervals *Xgwm639a-Xgwm639c* and *Xgwm234a-XDuPw115a*, respectively [51]. *QYr*.*caas-5BL*.*1* and *QYr*.*caas-5BL*.*2* in Libellula were mapped in the *Xwmc415-Xwmc537* and *Xbarc142-Xgwm604* marker intervals, respectively [62]. *QYr*.*caas-5BL*.*3* in Shanghai 3/Catbird was reported on chromosome 5BL between *Xwmc75* and *Xbarc275*, a position similar to that of *QYr*.*caas-5BL*.*2* [46]. Based on the consensus SSR map [21], *QYrdr*.*wgp-5BL*.*2* close to SSR marker *Xgwm499* in the present study should be in the same region as *QYr*.*jirc-5BL*, *QYr*.*caas-5BL*.*1* and *QYr*.*inra-5BL*.*1*. Because these QTL all confer adult-plant resistance and their wheat carriers are all of European origin, they are likely the same. Both *QYr*.*caas-5BL*.*2* and *QYr*.*caas-5BL*.*3* should be distal to *QYrdr*.*wgp-5BL*.*2* according to the consensus map [21].

### QYrdr.wgp-5DL

*QYrdr*.*wgp-5DL* confers an all-stage resistance in Druchamp to old races like PST-35 and PST-45. Chen et al. [53] located *YrDa2* to chromosome 5D in US cultivar Daws using monosomic analysis, but its specific chromosomal location is unknown. Because Daws is susceptible to PST-35 and PST-45, while *QYrdr*.*wgp-5DL* is effective against the two races, the two genes should be different, and *QYrdr*.*wgp-5DL* should be a new gene, since no any other stripe rust resistance QTL have been reported on 5DL [4], [34].

### *QYrdr*.*wgp-6BL*.*1* and *QYrdr*.*wgp-6BL*.*2*

In the present study, we mapped two QTL on chromosome 6BL. *QYrdr*.*wgp-6BL*.*1* confers all-stage resistance and *QYrdr*.*wgp-6BL*.*2* confers HTAP resistance. These QTL were about 22 cM apart. Because only SNP markers were identified for *QYrdr*.*wgp-6BL*.*1*, its relationships to previously reported stripe rust genes could not be determined.

Several stripe rust resistance genes have been mapped on chromosome 6BS, such as *Yr35* [63], *Yr36* [64], an Oligoculm QTL [35], *QYrst*.*wgp-6BS*.*1* and *QYrst*.*wgp-6BS*.*2* [65], *QYr*.*sun-6B* [66], *QYr*.*caas-6BS* [57] and *QYr*.*caas-6BS*.*2* [46], but only few on 6BL. Because *QYr*.*inra-6B* was mapped with markers in both 6BS and 6BL [67], it could be in the centromeric region. Christiansen et al. [48] reported a QTL in the population of a cross between Deben and Wasmo on chromosome 6BL linked to SSR markers *Xwmc397* and *Xwmc105b*. William et al. [36] mapped *QYr*.*cimmyt-6BL* in Pavon 76 to 6BL with SSR markers *Xgwm58* and *Xgwm626*. Because the QTL reported by Christiansen et al. [48] and William et al. [36] are both distal to *Xgwm626*, and *QYrdr*.*wgp-6BL*.*2* is proximal to that marker and closely linked to *Xgwm608* (Fig 4), the QTL in the present study should be different. Rosewarne et al. [68] mapped a QTL in Pastor on 6BL. As this QTL is close to SSR marker *Xgwm219*, it is more distal than either *QYr*.*cimmyt-6BL* or *QYrdr*.*wgp-6BL*.*2* on 6BL. Therefore, *QYrdr*.*wgp-6BL*.*2* is likely a new gene for stripe rust resistance.

### Non-race specificity of HTAP resistance in Druchamp

Druchamp and Michigan Amber were included in a stripe rust monitoring nursery that has been evaluated every year at Pullman and Mt. Vernon in the State of Washington and other locations across the US for more than 40 years, and Druchamp has always shown a high level of resistance. In the present study, the F_8_ RIL population was evaluated at the two locations for a period spanning six years from 2005 to 2011, during which 6–21 races were detected each year in these two locations with the presence of races virulent on the seedlings of Druchamp [16], [45], [69]. During the field experiments at the two locations in 2010 and 2011, stripe rust samples were collected from some of the RILs, and all samples were identified as races virulent on the seedlings of Druchamp (data not shown). The virulence data of the *Pst* populations indicated that the resistant phenotypes observed in the fields were controlled by QTL for the HTAP resistance, but not for the all-stage resistance in Druchamp. This is supported by the findings that none of the three race-specific all-stage resistance QTL was detected in the field experiments and that none of the eight QTL detected in the field experiments contributed to any of the observed seedling resistance in the greenhouse experiments. Most of the QTL for HTAP resistance identified in this study were detected in all field experiments and five of the eight HTAP QTL did not show significant interactions with the environments, indicating the race non-specificity nature of the resistance. The observed variations in individual gene effects in the experiments were mostly due to environmental effects as HTAP resistance is affected by temperature, moisture and disease pressure [1], [4].

## Conclusions

This study demonstrates the durability of HTAP resistance, and that the high level of HTAP resistance in Druchamp is controlled by a large number of QTL with various degrees of effectiveness. Among the total of 11 QTL detected in Druchamp, at least three (*QYrdr*.*wgp-5DL* for race-specific all-stage resistance and *QYrdr*.*wgp-3AL* and *QYrdr*.*wgp-6BL*.*2* for race non-specific HTAP resistance) are new. The study establishes the presence of *Yr29* in Druchamp. The QTL for either all-stage resistance or HTAP resistance are mostly recessive as the distribution of stripe rust responses of the F_8_ RILs were skewed more toward susceptibility. Because the HTAP QTL mostly had additive effects, RILs with more QTL displayed higher levels of resistance (Fig 5). The results of the present study and previous studies indicate that high level of HTAP resistance can be controlled by one, few or a large number of QTL [4]. Although the durability of stripe rust resistance in some other wheat cultivars is more dependent on the type of resistance and less dependent on the number of genes [4], [70], the durability of the resistance in Druchamp may be due to both the HTAP type and the large number of QTL. More studies are needed to elucidate the molecular mechanisms of durable type resistance. Practically, QTL for resistance that has been proven to be durable like the HTAP resistance in Druchamp should be valuable for developing wheat cultivars with durable resistance to stripe rust. Molecular markers linked to the QTL can be useful for marker-assisted selection. Multiple markers should be used as most of the closely linked markers for the QTL identified in this study are SNPs.

## Supporting Information

S1 Dataset Infection type data of seedling tests in the greenhouse. Infection types produced by *Pst* races on F_8_ lines derived from the Druchamp × Michigan Amber cross tested in seedling stage under low temperature (4–20^°^C) in the greenhouse.
(XLSX)

S2 Dataset Infection type and severity data of adult-plants tested in the greenhouse. Infection type and severity (%) produced by *Pst* races PST-25 (Table A in S2 Dataset) and PST-127 (Table B in S2 Dataset) on F_8_ lines derived from the Druchamp × Michigan Amber cross tested in seedling stage under low temperature (10–30^°^C) in the greenhouse.
(XLSX)

S3 Dataset Infection type and severity data of adult-plants tested in fields. Infection type and severity (%) of F_8_ lines derived from the Druchamp × Michigan Amber cross tested in Mount Vernon, WA 2005 (Table A in S3 Dataset), Pullman, WA 2006 (Table B in S3 Dataset), Mt. Vernon 2010 (Table C in S3 Dataset), Pullman 2010 (Table D in S3 Dataset), Mt. Vernon 2011 (Table E in S3 Dataset) and Pullman 2010 (Table F in S3 Dataset) under natural infection of *Puccinia striiformis* f. sp. *tritici*.
(XLSX)

S4 Dataset SSR markers. Alleles of 156 polymorphic SSR markers of the Druchamp × Michigan Amber F_8_ lines.
(XLSX)

S5 Dataset SNP markers. Alleles of 2,535 polymorphic SNP markers of the Druchamp × Michigan Amber F_8_ lines.
(XLSX)

S6 Dataset Linkage groups constructed for the Druchamp X Michigan Amber F_8_ population using SSR and SNP markers. A total of 32 linkage groups representing 18 chromosomes constructed with 156 SSR and 2,535 SNP markers and their map positions and alleles in the Druchamp × Michigan Amber F_8_ lines.
(XLSX)

S1 Table Effects of different combinations of the QTL. Mean rAUDPC and IT in six field experiments (Pullman 2006, 2010 and 2011 and Mt. Vernon 2005, 2010 and 2011) in the Druchamp × Michigan Amber RIL population.
(DOCX)

## References

[1] Chen XM (2005) Epidemiology and control of stripe rust [*Puccinia striiformis* f. sp. *tritici*] on wheat. Can J Plant Pathol 27:314–337.

[2] Stubbs RW (1985) Stripe rust In: Roelfs AP, Bushnell WR (eds) The Cereal Rusts. Vol. II, Disease, Distribution, Epidemiology and Control Academic Press, Orlando, 61–101.

[3] Wellings CR (2011) Global status of stripe rust: a review of historical and current threats. Euphytica 179:129–141.

[4] Chen XM (2013) Review article: High-temperature adult-plant resistance, key for sustainable control of stripe rust. Amer J Plant Sci 4:608–627.

[5] Chen XM (2014) Integration of cultivar resistance and fungicide application for control of wheat stripe rust. Can J Plant Pathol 36:311–326.

[6] Line RF (2002) Stripe rust of wheat and barley in North America: a retrospective historical review. Annu Rev Phytopathol 40:75–118. 12147755 10.1146/annurev.phyto.40.020102.111645

[7] Qayoum A, Line RF (1985) High-temperature, adult-plant resistance to stripe rust of wheat. Phytopathology 75:1121–1125.

[8] Line RF, Chen XM (1995) Successes in breeding for and managing durable resistance to wheat rusts. Plant Dis 79:1254–1255.

[9] Line RF, Qayoum A (1992) Virulence, aggressiveness, evolution, and distribution of races of *Puccinia striiformis* (the cause of stripe rust of wheat) in North America 1968–1987. US Department of Agriculture Technical Bulletin No. 1788, p44.

[10] Chen XM, Line RF (1992) Inheritance of stripe rust resistance in wheat cultivars used to differentiate races of *Puccinia striiformis* in North America. Phytopathology 82:633–637.

[11] Chen XM, Line RF (1992) Identification of stripe rust resistance genes in wheat genotypes used to differentiate North American races of *Puccinia striiformis* . Phytopathology 82:1428–1434.

[12] Chen XM, Line RF (1995) Gene action in wheat cultivars for durable, high-temperature, adult-plant resistance and interaction with race-specific, seedling resistance to *Puccinia striiformis* . Phytopathology 85:567–572.

[13] Chen XM, Line RF (1995) Gene number and heritability of wheat cultivars with durable, high-temperature, adult-plant (HTAP) resistance and interaction of HTAP and race-specific seedling resistance to *Puccinia striiformis* . Phytopathology 85:573–578.

[14] Chen XM, Jones SS, Line RF (1996) Chromosomal location of genes for resistance to *Puccinia striiformis* in seven wheat cultivars with resistance genes at the *Yr3* and *Yr4* loci. Phytopathology 86:1228–1233.

[15] Chen XM, Moore M, Milus EA, Long DL, Line RF, Marshall D, et al (2002) Wheat stripe rust epidemics and races of *Puccinia striiformis* f. sp. *tritici* in the United States in 2000. Plant Dis 86:39–46. 10.1094/PDIS.2002.86.1.39 30822996

[16] Chen XM, Penman L, Wan AM, Cheng P (2010) Virulence races of *Puccinia striiformis* f. sp. *tritici* in 2006 and 2007 and development of wheat stripe rust and distributions, dynamics, and evolutionary relationships of races from 2000 to 2007 in the United States. Can J Plant Pathol 32:315–333.

[17] Sharma-Poudyal D, Chen XM, Rupp R (2014) Potential oversummering and overwintering regions for the wheat stripe rust pathogen in the contiguous United States. Int J Biometeorol 58:987–997. 10.1007/s00484-013-0683-6 23722926

[18] Lin F, Chen XM (2007) Genetics and molecular mapping of genes for race-specific all-stage resistance and non-race specific high-temperature adult-plant resistance to stripe rust in spring wheat cultivar Alpowa. Theor Appl Genet 114:1277–1287. 17318493 10.1007/s00122-007-0518-0

[19] Clarke JD (2002) Cetyltrimethyl ammonium bromide (CTAB) DNA miniprep for plant DNA isolation Arabidopsis: A Laboratory Manual (eds. Weigel and Glazebrook). CSHL Press Cold Spring Harbor, NY, USA. 10.1101/pdb.prot5177 20147112

[20] Maniatis TA, Frisch EF, Sambrook J (1982) Molecular cloning: a laboratory manual Cold Spring Harbor Laboratory, Cold Spring Harbor 5.14–5.17.

[21] Somers DJ, Isaac P, Edwards K (2004) A high-density microsatellite consensus map for bread wheat (*Triticum aestivum* L.). Theor Appl Genet 109:1105–1114. 15490101 10.1007/s00122-004-1740-7

[22] Schuelke M (2000) An economic method for the fluorescent labeling of PCR fragments. Nature Biotechnol 18:233–234. 10657137 10.1038/72708

[23] Cavanagh CR, Chao SM, Wang SC, Huang BE, Stephen S, Kiani S, et al (2013) Genome-wide comparative diversity uncovers multiple targets of selection for improvement in hexaploid wheat landraces and cultivars. Proc Natl Acad Sci USA 110:8057–8062. 10.1073/pnas.1217133110 23630259 PMC3657823

[24] Van Ooijen JW (2006) JoinMap 4, software for the calculation of genetic linkage maps in experimental populations. Kyazma B.V., Wageningen, Netherlands.

[25] Kosambi DD (1944) The estimation of map distances from recombination values. Ann Eugen 12:172–175.

[26] Sourdille P, Singh S, Cadalen T, Brown-Guedira GL, Gay G, Qi L, et al (2004) Microsatellite-based deletion bin system for the establishment of genetic-physical map relationships in wheat (*Triticum aestivum* L.). Funct Integr Genomics 4:12–25. 15004738 10.1007/s10142-004-0106-1

[27] Voorrips RE (2002) MapChart: Software for the graphical presentation of linkage maps and QTLs. J Hered 93:77–78. 12011185 10.1093/jhered/93.1.77

[28] Zeng ZB (1993) Theoretical basis for separation of multiple linked gene effects in mapping quantitative trait loci. Proc Natl Acad Sci USA 90:10972–10976. 8248199 10.1073/pnas.90.23.10972 PMC47903

[29] Zeng ZB (1994) Precision mapping of quantitative trait loci. Genetics 136:1457–1458. 8013918 10.1093/genetics/136.4.1457 PMC1205924

[30] Wang S, Basten CJ, Zeng Z-B (2007) Windows QTL Cartographer 25. Department of Statistics. North Carolina State University, Raleigh, NC, USA.

[31] Churchill G, Doerge RW (1994) Empirical threshold values for quantitative trait mapping. Genetics 138:963–971. 7851788 10.1093/genetics/138.3.963 PMC1206241

[32] Tang S, Leon A, Bridges WC, Knapp SJ (2006) Quantitative trait loci for genetically correlated seed traits are tightly linked to branching and pericarp pigment loci in sunflower. Crop Sci 46:721–734.

[33] Lillemo M, Asalf B, Singh RP, Huerta-Espino J, Chen XM, He ZH, et al (2008) The adult plant rust resistance loci *Lr34/Yr18* and *Lr46/Yr29* are important determinants of partial resistance to powdery mildew in bread wheat line Saar. Theor Appl Genet 116:1155–1166. 10.1007/s00122-008-0743-1 18347772

[34] Rosewarne GM, Herrera-Foessel SA, Singh RP, Huerta‑Espino J, Lan CX, He ZH (2013) Quantitative trait loci of stripe rust resistance in wheat. Theor Appl Genet 126:2427–2449. 10.1007/s00122-013-2159-9 23955314 PMC3782644

[35] Suenaga K, Singh RP, Huerta-Espino J, William HM (2003) Microsatellite markers for genes *Lr34/Yr18* and other quantitative trait loci for leaf rust and stripe rust resistance in bread wheat. Phytopathology 93:881–890. 10.1094/PHYTO.2003.93.7.881 18943170

[36] William HM, Singh RP, Huerta-Espino J, Palacios G, Suenaga K (2006) Characterization of genetic loci conferring adult plant resistance to leaf rust and stripe rust in spring wheat. Genome 49:977–990. 17036073 10.1139/g06-052

[37] Rosewarne GM, Singh RP, Huerta-Espino J, William HM, Bouchet S, Cloutier S, et al (2006) Leaf tip necrosis, molecular markers and β1-proteasome subunits associated with the slow rusting resistance genes *Lr46/Yr29* . Theor Appl Genet 112:500–508. 16331478 10.1007/s00122-005-0153-6

[38] Melichar JPE, Berry S, Newell C, MacCormack R, Boyd LA (2008) QTL identification and microphenotype characterization of the developmentally regulated yellow rust resistance in the UK wheat cultivar Guardian. Theor Appl Genet 117:391–399. 10.1007/s00122-008-0783-6 18481042

[39] Fang HL, Yong CN, Hui D, Gen JT (2007) Mapping of a major stripe rust resistance gene in Chinese native wheat variety Chike using microsatellite markers. J Genet Genomics 34:1123–1130. 18155625 10.1016/S1673-8527(07)60128-3

[40] Lin F, Chen XM (2008) Molecular mapping of genes for race-specific overall resistance to stripe rust in wheat cultivar Express. Theor Appl Genet 116:797–806. 10.1007/s00122-008-0713-7 18214420

[41] Lin F, Chen XM (2009) Quantitative trait loci for non-race-specific, high-temperature adult-plant resistance to stripe rust in wheat cultivar Express. Theor Appl Genet 118:631–642. 10.1007/s00122-008-0894-0 18815766

[42] Li Q, Hu ML, Chen J, Jing JX, Wang BT, Zhou X (2010) Inheritance and molecular mapping of genes for all-stage resistance to stripe rust in wheat cultivar N. Strampelli. Can J Plant Sci 90:529–536.

[43] Hou L, Ma DF, Hu ML, He MM, Lu Y, Jing JX (2013) Genetic analysis and molecular mapping of an all-stage stripe rust resistance gene in *Triticum aestivum—Haynaldia villosa* translocation line V3. J. Integ. Agri. 12:2197–2208.

[44] Sharma-Poudyal D, Chen XM, Wan AM, Zhan GM, Kang ZS, Cao SQ, et al (2013) Virulence characterization of international collections of the wheat stripe rust pathogen, *Puccinia striiformis* f. sp. *tritici* . Plant Dis 97:379–386. 30722363 10.1094/PDIS-01-12-0078-RE

[45] Wan AM, Chen XM (2014) Virulence characterization of *Puccinia striiformis* f. sp. *tritici* using a new set of *Yr* single-gene line differentials in the United States in 2010. Plant Dis 98:1534–1542. 30699782 10.1094/PDIS-01-14-0071-RE

[46] Ren Y, He ZH, Li J, Lillemo M, Wu L (2012) QTL mapping of adult-plant resistance to stripe rust in a population derived from common wheat cultivars Naxos and Shanghai 3/Catbird. Theor Appl Genet 125:1211–1221. 10.1007/s00122-012-1907-6 22798057

[47] McIntosh RA, Yamazaki Y, Dubcovsky J, Rogers J, Morris C, Appels R, et al. (2013) Catalogue of gene symbols for wheat. 12th Int Wheat Genet Symp, 8–13 Sep 2013, Yokohama, Japan. Available: http://www.shigen.nig.ac.jp/wheat/komugi/genes/download.jsp. Accessed 2014 Nov 10.

[48] Christiansen MJ, Feenstra B, Skovgaard IM, Andersen SB (2006) Genetic analysis of resistance to yellow rust in hexaploid wheat using a mixture model for multiple crosses. Theor Appl Genet 112:581–591. 16395570 10.1007/s00122-005-0128-7

[49] Rosewarne GM, Singh RP, Huerta-Espino J, Rebetzke GJ (2008) Quantitative trait loci for slow-rusting resistance in wheat to leaf rust and stripe rust identified with multi-environment analysis. Theor Appl Genet 116:1027–1034. 10.1007/s00122-008-0736-0 18335201

[50] Boukhatem N, Baret PV, Mingeot D, Jacquemin JM (2002) Quantitative trait loci for resistance against yellow rust in two wheat-derived recombinant inbred line populations. Theor Appl Genet 104:111–118. 12579435 10.1007/s001220200013

[51] Mallard S, Gaudet D, Aldeia A, Abelard C, Besnard AL, Sourdille P, et al (2005) Genetic analysis of durable resistance to yellow rust in bread wheat. Theor Appl Genet 110:1401–1409. 15841362 10.1007/s00122-005-1954-3

[52] Guo Q, Zhang ZJ, Xu YB, Li GH, Feng J, Zhou Y (2008) Quantitative trait loci for high-temperature adult-plant and slow-rusting resistance to *Puccinia striiformis* f. sp. *tritici* in wheat cultivars. Phytopathology 98:803–809. 10.1094/PHYTO-98-7-0803 18943256

[53] Chen XM, Line RF, Jones SS (1995) Chromosomal location of genes for resistance to *Puccinia striiformis* in winter wheat cultivars Heines VII, Clement, Moro, Tyee, Tres, and Daws. Phytopathology 85:1362–1367.

[54] Cao X, Zhou J, Gong X, Zhao G, Jia J, Qi X (2012) Identification and validation of a major quantitative trait locus for slow-rusting resistance to stripe rust in wheat. J Integ Plant Biol 54:330–344. 10.1111/j.1744-7909.2012.01111.x 22349012

[55] Bariana HS, Parry N, Barclay IR, Loughman R, McLean RJ, Shankar M, et al (2006) Identification and characterization of stripe rust resistance gene *Yr34* in common wheat. Theor Appl Genet 112:1143–1148. 16435125 10.1007/s00122-006-0216-3

[56] Chhuneja P, Kaur S, Garg T, Ghai M, Kaur S, Prashar M, et al (2008) Mapping of adult plant stripe rust resistance genes in diploid A genome wheat species and their transfer to bread wheat. Theor Appl Genet 116:313–324. 17989954 10.1007/s00122-007-0668-0

[57] Lan CX, Liang SS, Zhou XC, Zhou G, Lu QL, Xia XC, et al (2010) Identification of genomic regions controlling adult-plant stripe rust resistance in Chinese landrace Pingyuan 50 through bulked segregant analysis. Phytopathology 100:313–318. 10.1094/PHYTO-100-4-0313 20205534

[58] Lowe I, Jankuloski L, Chao SM, Chen XM, See DR, Dubcovsky J (2011) Mapping and validation of QTL which confer partial resistance to broadly virulent post-2000 North American races of stripe rust in hexaploid wheat. Theor Appl Genet 123:143–157. 10.1007/s00122-011-1573-0 21455722 PMC4761445

[59] Jagger LJ, Newell C, Berry ST, MacCormack R, Boyd LA (2011) The Genetic characterisation of stripe rust resistance in the German wheat cultivar Alcedo. Theor Appl Genet 122:723–733. 10.1007/s00122-010-1481-8 21076811

[60] Worland AJ (1988) Studies of the resistance of wheat to yellow rust. 1987 Annual Report, pp. 8–9. Institute of Plant Science Research, Cambridge.

[61] Yang MN, Xu ZB, Wang MN, Song JR, Jing JX, Li ZQ (2008) Inheritance and molecular mapping of stripe rust resistance gene *Yr88375* in Chinese wheat line Zhongliang 88375. Scientia Agric Sinica 41:2931–2936.

[62] Lu YM, Lan CX, Liang SS, Zhou XC, Liu D, Zhou G, et al (2009) QTL mapping for adult-plant resistance to stripe rust in Italian common wheat cultivars Libellula and Strampelli. Theor Appl Genet 119:1349–1359. 10.1007/s00122-009-1139-6 19756474

[63] Dadkhodaie NA, Karaoglou H, Wellings CR, Park RF (2011) Mapping genes *Lr53* and *Yr35* on the short arm of chromosome 6B of common wheat with microsatellite markers and studies of their associate with *Lr36* . Theor Appl Genet 122:479–487. 10.1007/s00122-010-1462-y 20924745

[64] Uauy C, Brevis JC, Chen XM, Khan IA, Jackson LF, Chicaiza O, et al (2005) High-temperature adult plant (HTAP) stripe rust resistance gene *Yr36* from *Triticum turgidum* ssp. *dicoccoides* is closely linked to the grain protein content locus *Gpc-B1* . Theor Appl Genet 112:97–105. 16208504 10.1007/s00122-005-0109-x

[65] Santra DK, Chen XM, Santra M, Campbell KG, Kidwell KK (2008) Identification and mapping QTL for high-temperature adult-plant resistance to stripe rust in winter wheat (*Triticum aestivum* L.) cultivar ‘Stephens’. Theor Appl Genet 117:793–802. 10.1007/s00122-008-0820-5 18584147

[66] Bariana HS, Bansal UK, Schmidt A, Lehmensiek A, Kaur J, Miah H, et al (2010) Molecular mapping of adult plant stripe rust resistance in wheat and identification of pyramided QTL genotypes. Euphytica 176:251–260.

[67] Dedryver F, Paillard S, Mallard S, Robert O, Trottet M, Negre S, et al (2009) Characterization of genetic components involved in durable resistance to stripe rust in the bread wheat ‘Renan’. Phytopathology 99:968–973. 10.1094/PHYTO-99-8-0968 19594316

[68] Rosewarne GM, Singh RP, Huerta-Espino J, Herrera-Foessel SA, Forrest KL, Hayden MJ, et al (2012) Analysis of leaf and stripe rust severities reveals pathotype changes and multiple minor QTLs associated with resistance in an Avocet × Pastor wheat population. Theor Appl Genet 124:1283–1294. 10.1007/s00122-012-1786-x 22274764

[69] Wan AM, Chen XM (2012) Virulence, frequency, and distribution of races of *Puccinia striiformis* f. sp. *tritici* and *P*. *striiformis* f. sp. *hordei* identified in the United States in 2008 and 2009. Plant Dis 96:67–74. 30731853 10.1094/PDIS-02-11-0119

[70] Chen XM, Coram T, Huang XL, Wang MN, Dolezal A (2013) Understanding molecular mechanisms of durable and non-durable resistance to stripe rust in wheat using a transcriptomics approach. Curr Genom 14:111–126. 10.1186/gb-2013-14-4-111 24082821 PMC3637676

