# Supplementary material for: Mapping a Large Number of QTL for Durable Resistance to Stripe Rust in Winter Wheat Druchamp Using SSR and SNP Markers
Source: PLoS One. 2015 May 13;10(5):e0126794. doi: 10.1371/journal.pone.0126794 (PMC4430513; doi:10.1371/journal.pone.0126794)
Supplement: S1 Table — Mean rAUDPC and IT in six field experiments (Pullman 2006, 2010 and 2011 and Mt. Vernon 2005, 2010 and 2011) in the Druchamp × Michigan Amber RIL population. (DOCX) [file pone.0126794.s007.docx]

**S1 Table.**  **Effects of different combinations the number of the QTL in combination in the F8 RILs of the Druchamp × Michigan Amber RIL population determined based on mean rAUDPC) and IT in six field experiments (Pullman 2006, 2010 and 2011 and Mt. Vernon (Mt.V.) 2005, 2010 and 2011).**

| **No. of** |  | **No. of** |  | **Mean rAUDPC (%)** | | |  | **Mean IT** | | |
| --- | --- | --- | --- | --- | --- | --- | --- | --- | --- | --- |
| **QTL** | **Single or combination of QTL** | **RILs** | **RIL** | **Pull.** | **Mt.V.** | **Mean** |  | **Pull.** | **Mt.V.** | **Mean** |
| 1 | 5AL | 1 | F_8_-14 | 81.4 | 70.8 | 76.1 |  | 8.3 | 7.6 | 7.9 |
| 2 | 5BL+6BL |  | F_8_-25 | 89.9 | 100.2 | 95.1 |  | 8.1 | 8.2 | 8.2 |
| 2 | 1DS+6BL |  | F_8_-36 | 85.1 | 83.9 | 84.5 |  | 8.0 | 7.5 | 7.8 |
| 2 | 1BL.2+5AL |  | F_8_-46 | 71.0 | 62.7 | 66.9 |  | 7.8 | 7.5 | 7.6 |
| 2 | 1BL.2+6BL |  | F_8_-59 | 55.8 | 71.6 | 63.7 |  | 6.7 | 6.9 | 6.8 |
| 2 | 1BL.1+6BL |  | F_8_-66 | 75.3 | 84.8 | 80.0 |  | 7.3 | 6.8 | 7.0 |
| 2 | 5BL+6BL |  | F_8_-80 | 81.1 | 91.9 | 86.5 |  | 7.8 | 8.1 | 8.0 |
| 2 | 1BL.1+2BL |  | F_8_-83 | 75.3 | 57.4 | 66.3 |  | 7.7 | 5.8 | 6.8 |
| 2 | 1BL.2+5AL |  | F_8_-94 | 86.9 | 63.2 | 75.0 |  | 7.6 | 7.2 | 7.4 |
| 2 | 3AL+5BL |  | F_8_-95 | 78.1 | 85.8 | 82.0 |  | 7.5 | 7.6 | 7.5 |
| 2 | 1DS+3AL |  | F_8_-100 | 97.7 | 76.8 | 87.2 |  | 8.3 | 7.9 | 8.1 |
|  | **Mean** | **10** |  | **79.6** | **77.8** | **78.7** |  | **7.7** | **7.3** | **7.5** |
| 3 | 1BL.2+1BL.1+5AL |  | F_8_-3 | 58.2 | 54.0 | 56.1 |  | 7.1 | 6.0 | 6.5 |
| 3 | 1BL.1+1DS+2BL |  | F_8_-18 | 83.2 | 54.9 | 69.1 |  | 8.2 | 6.3 | 7.3 |
| 3 | 1BL.1+5AL+6BL |  | F_8_-33 | 70.3 | 64.4 | 67.3 |  | 7.9 | 7.4 | 7.7 |
| 3 | 1BL.1+1DS+5AL |  | F_8_-35 | 56.0 | 51.5 | 53.7 |  | 6.8 | 5.5 | 6.1 |
| 3 | 1BL.1+5AL+6BL |  | F_8_-40 | 62.2 | 56.3 | 59.2 |  | 7.2 | 6.2 | 6.7 |
| 3 | 1BL.1+1DS+5AL |  | F_8_-45 | 58.0 | 47.0 | 52.5 |  | 5.8 | 4.6 | 5.2 |
| 3 | 1BL.2+2BL+5AL |  | F_8_-61 | 74.6 | 58.6 | 66.6 |  | 7.7 | 7.0 | 7.3 |
| 3 | 1BL.2+1DS+5AL |  | F_8_-69 | 53.0 | 39.3 | 46.2 |  | 6.6 | 4.2 | 5.4 |
| 3 | 1BL.1+5AL+6BL |  | F_8_-77 | 54.7 | 44.3 | 49.5 |  | 6.3 | 5.3 | 5.8 |
| 3 | 1DS+5BL+6BL |  | F_8_-79 | 56.9 | 48.0 | 52.5 |  | 7.5 | 4.7 | 6.1 |
| 3 | 1DS+2BL+5AL |  | F_8_-84 | 86.5 | 63.6 | 75.0 |  | 8.1 | 6.7 | 7.4 |
| 3 | 5AL+5BL+6BL |  | F_8_-85 | 48.3 | 49.6 | 48.9 |  | 6.5 | 5.6 | 6.0 |
| 3 | 5AL+5BL+6BL |  | F_8_-86 | 44.1 | 60.5 | 52.3 |  | 6.0 | 6.5 | 6.3 |
| 3 | 1BL.1+1DS+6BL |  | F_8_-99 | 64.4 | 49.9 | 57.2 |  | 7.6 | 5.6 | 6.6 |
| 3 | 3AL+5BL+6BL |  | F_8_-13 | 54.2 | 63.9 | 59.1 |  | 7.7 | 7.3 | 7.5 |
| 3 | 1BL.1+3AL+5BL |  | F_8_-24 | 79.0 | 68.4 | 73.7 |  | 7.9 | 7.5 | 7.7 |
| 3 | 1BL.1+1DS+3AL |  | F_8_-29 | 88.1 | 61.2 | 74.6 |  | 7.9 | 6.4 | 7.2 |
| 3 | 3AL+5AL+5BL |  | F_8_-32 | 60.6 | 54.5 | 57.6 |  | 7.3 | 5.2 | 6.2 |
| 3 | 1BL.2+1BL.1+3AL |  | F_8_-62 | 45.7 | 53.1 | 49.4 |  | 7.5 | 6.5 | 7.0 |
| 3 | 1BL.2+1DS+3AL |  | F_8_-63 | 62.1 | 54.6 | 58.3 |  | 7.8 | 5.8 | 6.8 |
| 3 | 1BL.2+1BL.1+3AL |  | F_8_-71 | 51.3 | 54.0 | 52.6 |  | 7.8 | 7.4 | 7.6 |
| 3 | 1BL.2+3AL+5BL |  | F_8_-41 | 44.3 | 56.0 | 50.1 |  | 6.7 | 6.6 | 6.7 |
|  | **Mean** | **22** |  | **61.6** | **54.9** | **58.3** |  | **7.3** | **6.1** | **6.7** |
| 4 | 2BL+5AL+5BL+6BL |  | F_8_-5 | 55.5 | 54.4 | 55.0 |  | 6.7 | 5.7 | 6.2 |
| 4 | 1BL.2+2BL+5AL+6BL |  | F_8_-7 | 46.3 | 35.8 | 41.1 |  | 6.9 | 5.0 | 5.9 |
| 4 | 1BL.2+1DS+2BL+6BL |  | F_8_-8 | 47.6 | 37.3 | 42.4 |  | 7.2 | 5.1 | 6.2 |
| 4 | 1BL.2+1BL.1+5AL+5BL |  | F_8_-22 | 48.7 | 55.6 | 52.2 |  | 6.8 | 6.2 | 6.5 |
| 4 | 1DS+5AL+5BL+6BL |  | F_8_-39 | 59.5 | 60.6 | 60.1 |  | 5.4 | 6.2 | 5.8 |
| 4 | 1BL.1+1DS+2BL+6BL |  | F_8_-47 | 69.8 | 59.0 | 64.4 |  | 7.9 | 5.7 | 6.8 |
| 4 | 1BL.2+2BL+5AL+6BL |  | F_8_-49 | 60.5 | 46.4 | 53.5 |  | 7.5 | 5.2 | 6.4 |
| 4 | 1DS+2BL+5BL+6BL |  | F_8_-50 | 46.3 | 53.8 | 50.1 |  | 7.3 | 6.2 | 6.7 |
| 4 | 1BL.2+2BL+5AL+6BL |  | F_8_-51 | 45.1 | 52.6 | 48.8 |  | 6.5 | 6.2 | 6.3 |
| 4 | 1BL.2+1BL.1+1DS+5AL |  | F_8_-57 | 44.0 | 48.1 | 46.1 |  | 7.1 | 4.5 | 5.8 |
| 4 | 1BL.2+1BL.1+5AL+6BL |  | F_8_-76 | 44.1 | 50.9 | 47.5 |  | 5.2 | 5.6 | 5.4 |
| 4 | 1BL.2+5AL+5BL+6BL |  | F_8_-89 | 58.9 | 54.4 | 56.6 |  | 7.7 | 6.2 | 6.9 |
| 4 | 1DS+5AL+5BL+6BL |  | F_8_-92 | 75.0 | 46.0 | 60.5 |  | 6.6 | 5.2 | 5.9 |
| 4 | 1BL.2+1BL.1+1DS+6BL |  | F_8_-93 | 82.3 | 54.1 | 68.2 |  | 8.0 | 6.3 | 7.1 |
| 4 | 1BL.1+2BL+5AL+5BL |  | F_8_-98 | 49.1 | 44.1 | 46.6 |  | 5.8 | 4.4 | 5.1 |
| 4 | 1BL.2+2BL+3AL+5BL |  | F_8_-6 | 35.7 | 39.4 | 37.5 |  | 5.7 | 4.3 | 5.0 |
| 4 | 1BL.2+1DS+2BL+3AL |  | F_8_-11 | 57.7 | 50.2 | 53.9 |  | 7.6 | 5.6 | 6.6 |
| 4 | 1DS+3AL+5AL+5BL |  | F_8_-17 | 58.1 | 36.1 | 47.1 |  | 6.9 | 4.2 | 5.6 |
| 4 | 1BL.1+2BL+3AL+5BL |  | F_8_-19 | 64.2 | 38.3 | 51.3 |  | 6.8 | 4.7 | 5.8 |
| 4 | 1BL.1+2BL+3AL+5AL |  | F_8_-34 | 84.5 | 59.4 | 71.9 |  | 8.0 | 6.7 | 7.4 |
| 4 | 1BL.1+3AL+5BL+6BL |  | F_8_-72 | 72.0 | 58.0 | 65.0 |  | 7.1 | 6.0 | 6.6 |
| 4 | 1DS+3AL+5AL+5BL |  | F_8_-96 | 84.5 | 69.0 | 76.8 |  | 8.0 | 7.0 | 7.5 |
|  | **Mean** | **22** |  | **58.6** | **50.2** | **54.4** |  | **6.9** | **5.6** | **6.2** |
| 5 | 1BL.1+1DS+2BL+5AL+5BL |  | F_8_-12 | 59.6 | 41.1 | 50.3 |  | 7.5 | 4.6 | 6.0 |
| 5 | 1BL.2+1BL.1+2BL+5AL+5BL |  | F_8_-15 | 36.1 | 35.4 | 35.7 |  | 6.1 | 3.8 | 5.0 |
| 5 | 1BL.2+1BL.1+1DS+5AL+6BL |  | F_8_-53 | 55.0 | 40.7 | 47.9 |  | 7.3 | 4.7 | 6.0 |
| 5 | 1BL.2+1DS+2BL+5AL+6BL |  | F_8_-56 | 38.5 | 40.7 | 39.6 |  | 5.4 | 4.4 | 4.9 |
| 5 | 1BL.1+1DS+5AL+5BL+6BL |  | F_8_-67 | 71.9 | 64.0 | 67.9 |  | 7.5 | 6.0 | 6.8 |
| 5 | 1BL.1+2BL+5AL+5BL+6BL |  | F_8_-73 | 73.5 | 66.6 | 70.0 |  | 7.1 | 6.1 | 6.6 |
| 5 | 1BL.1+2BL+5AL+5BL+6BL |  | F_8_-78 | 64.8 | 53.8 | 59.3 |  | 7.7 | 6.0 | 6.8 |
| 5 | 1BL.2+1DS+2BL+5BL+6BL |  | F_8_-90 | 39.1 | 35.5 | 37.3 |  | 5.5 | 4.1 | 4.8 |
| 5 | 1BL.1+1DS+3AL+5AL+6BL |  | F_8_-1 | 41.3 | 34.4 | 37.9 |  | 5.4 | 4.0 | 4.7 |
| 5 | 1BL.2+1BL.1+1DS+3AL+5AL |  | F_8_-2 | 40.1 | 34.5 | 37.3 |  | 6.2 | 3.7 | 5.0 |
| 5 | 1BL.2+1BL.1+1DS+2BL+3AL |  | F_8_-23 | 52.4 | 47.2 | 49.8 |  | 7.0 | 5.2 | 6.1 |
| 5 | 1BL.2+1DS+3AL+5AL+5BL |  | F_8_-26 | 40.7 | 37.3 | 39.0 |  | 5.6 | 4.4 | 5.0 |
| 5 | 1BL.2+1BL.1+1DS+3AL+5AL |  | F_8_-42 | 36.1 | 42.5 | 39.3 |  | 6.6 | 4.8 | 5.7 |
| 5 | 1BL.2+1BL.1+3AL+5AL+6BL |  | F_8_-44 | 51.3 | 58.0 | 54.6 |  | 6.6 | 5.9 | 6.2 |
| 5 | 1BL.2+1DS+2BL+3AL+5BL |  | F_8_-48 | 55.8 | 49.2 | 52.5 |  | 7.9 | 5.4 | 6.6 |
| 5 | 1BL.2+2BL+3AL+5AL+6BL |  | F_8_-54 | 33.4 | 30.4 | 31.9 |  | 6.1 | 4.0 | 5.1 |
| 5 | 1BL.2+2BL+3AL+5AL+6BL |  | F_8_-64 | 31.1 | 47.0 | 39.1 |  | 4.5 | 4.9 | 4.7 |
| 5 | 1BL.1+3AL+5AL+5BL+6BL |  | F_8_-65 | 71.8 | 70.3 | 71.0 |  | 7.2 | 7.1 | 7.2 |
| 5 | 1BL.1+2BL+3AL+5BL+6BL |  | F_8_-81 | 58.8 | 44.6 | 51.7 |  | 6.3 | 5.1 | 5.7 |
| 5 | 1BL.2+1DS+3AL+5AL+6BL |  | F_8_-91 | 33.8 | 34.7 | 34.2 |  | 5.4 | 3.4 | 4.4 |
|  | **Mean** | **20** |  | **49.3** | **45.4** | **47.3** |  | **6.4** | **4.9** | **5.7** |
| 6 | 1BL.2+1DS+2BL+3AL+5AL+5BL |  | F_8_-4 | 14.9 | 20.9 | 17.9 |  | 2.9 | 3.0 | 3.0 |
| 6 | 1BL.2+1DS+2BL+3AL+5AL+5BL |  | F_8_-16 | 29.7 | 30.9 | 30.3 |  | 5.5 | 3.7 | 4.6 |
| 6 | 1BL.2+2BL+3AL+5AL+5BL+6BL |  | F_8_-20 | 18.6 | 30.9 | 24.7 |  | 3.3 | 3.7 | 3.5 |
| 6 | 1DS+2BL+3AL+5AL+5BL+6BL |  | F_8_-27 | 25.5 | 31.8 | 28.7 |  | 4.2 | 3.6 | 3.9 |
| 6 | 1BL.2+1BL.1+3AL+5AL+5BL+6BL |  | F_8_-28 | 35.0 | 45.7 | 40.4 |  | 5.7 | 5.4 | 5.6 |
| 6 | 1BL.2+1BL.1+1DS+2BL+3AL+5BL |  | F_8_-31 | 52.8 | 35.4 | 44.1 |  | 6.8 | 3.6 | 5.2 |
| 6 | 1BL.2+1BL.1+1DS+3AL+5AL+6BL |  | F_8_-52 | 46.8 | 40.3 | 43.6 |  | 5.8 | 4.8 | 5.3 |
| 6 | 1BL.2+1BL.1+1DS+2BL+3AL+5AL |  | F_8_-75 | 40.6 | 36.4 | 38.5 |  | 6.2 | 4.2 | 5.2 |
| 6 | 1BL.2+1BL.1+3AL+5AL+5BL+6BL |  | F_8_-87 | 25.2 | 36.9 | 31.0 |  | 4.2 | 3.9 | 4.1 |
| 6 | 1BL.2+1DS+2BL+3AL+5AL+6BL |  | F_8_-97 | 33.2 | 40.3 | 36.7 |  | 6.1 | 4.7 | 5.4 |
| 6 | 1BL.2+2BL+3AL+5AL+5BL+6BL |  | F_8_-43 | 44.9 | 47.5 | 46.2 |  | 4.9 | 5.5 | 5.2 |
|  | **Mean** | **11** |  | **33.4** | **36.1** | **34.7** |  | **5.1** | **4.2** | **4.6** |
| 7 | 1BL.2+1BL.1+1DS+2BL+3AL+5AL+6BL |  | F_8_-9 | 47.6 | 35.2 | 41.4 |  | 7.2 | 4.8 | 6.0 |
| 7 | 1BL.2+1BL.1+1DS+3AL+5AL+5BL+6BL |  | F_8_-21 | 27.6 | 32.8 | 30.2 |  | 4.9 | 4.6 | 4.8 |
| 7 | 1BL.2+1BL.1+1DS+2BL+3AL+5AL+5BL |  | F_8_-30 | 53.1 | 34.1 | 43.6 |  | 6.6 | 4.1 | 5.4 |
| 7 | 1BL.2+1DS+2BL+3AL+5AL+5BL+6BL |  | F_8_-37 | 28.6 | 20.0 | 24.3 |  | 3.4 | 2.8 | 3.1 |
| 7 | 1BL.2+1DS+2BL+3AL+5AL+5BL+6BL |  | F_8_-55 | 31.5 | 24.1 | 27.8 |  | 3.3 | 2.6 | 3.0 |
| 7 | 1BL.1+1DS+2BL+3AL+5AL+5BL+6BL |  | F_8_-68 | 27.8 | 35.3 | 31.5 |  | 4.3 | 3.7 | 4.0 |
| 7 | 1BL.2+1BL.1+1DS+3AL+5AL+5BL+6BL |  | F_8_-74 | 47.1 | 38.2 | 42.7 |  | 6.9 | 4.2 | 5.5 |
|  | **Mean** | **7** |  | **37.6** | **31.4** | **34.5** |  | **5.2** | **3.8** | **4.5** |
| 8 | 1BL.2+1BL.1+1DS+2BL+3AL+5AL+5BL+6BL | 1 | F_8_-38 | 20.4 | 20.9 | 20.7 |  | 2.7 | 2.5 | 2.6 |
| Druchamp | All QTL |  |  | 16.6 | 12.5 | 14.6 |  | 2.0 | 2.1 | 2.1 |
| Michigan Amber | No QTL |  |  | 100.0 | 100.0 | 100.0 |  | 8.3 | 8.3 | 8.3 |
